# Crystal-Storing Histiocytosis: The Iceberg of More Serious Conditions

**DOI:** 10.3390/diagnostics13020271

**Published:** 2023-01-11

**Authors:** Mousa Mobarki, Alexandra Papoudou-Bai, Jean Marc Dumollard, Abdulaziz H. Alhazmi, Shaqraa Musawi, Mohammed Ali Madkhali, Khalid Y. Muqri, Michel Péoc’h, Georgia Karpathiou

**Affiliations:** 1 Pathology Department, Faculty of Medicine, Jazan University, Jazan 45142, Saudi Arabia; 2 Pathology Department, Faculty of Medicine, University of Ioannina, 47100 Ioannina, Greece; 3 Pathology Department, University Hospital of Saint-Etienne, 42023 Saint-Etienne, France; 4 Microbiology and Parasitology Department, Faculty of Medicine, Jazan University, Jazan 45142, Saudi Arabia; 5 Department of Medical Laboratories Technology, College of Applied Medical Sciences, Jazan University, Jazan 45142, Saudi Arabia; 6 Division of Hematology and Oncology, Department of Internal Medicine, Faculty of Medicine, Jazan University, Jazan 45142, Saudi Arabia; 7 Faculty of Medicine, Jazan University, Jazan 45142, Saudi Arabia

**Keywords:** crystal storing histiocytosis, multiple myeloma, lymphoma, histiocytosis, eosinophilic, inclusions, macrophages

## Abstract

Crystal-storing histiocytosis is a rare condition that is histologically characterized by intracellular cytoplasmic crystalline inclusions. It usually presents monoclonal immunoglobulins that deposit within histiocytes, which accumulate and affect different organs of the human body and are commonly associated with lymphoproliferative conditions, especially those with plasmacytic differentiation. The prognosis of this condition is variable and related to the underlying clinical disease. In this review article, we aim to describe and discuss the clinical and pathological characteristics of crystal-storing histiocytosis based on the available literature and to provide a thorough differential diagnosis.

## 1. Introduction

**Figure 1:**
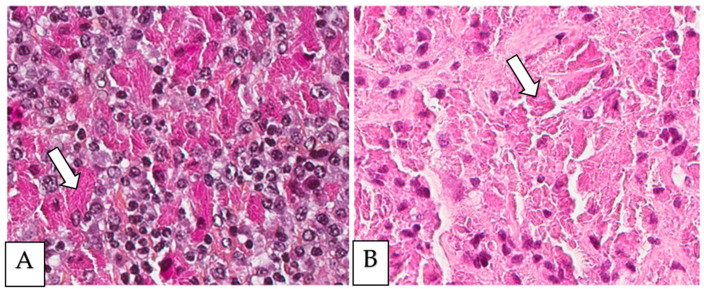
(**A**,**B**) Crystal-storing histiocytosis is characterized by an accumulation of histiocytes with intracytoplasmic crystalline inclusions (arrows). (**A**,**B**) High magnification of samples from two different cases. (**A**) A 70-year-old male who presented with abdominal pain, cervical lymphadenopathy, and weight loss with colonic white mucosal elevations on endoscopy. (**B**) A 50-year-old male with a suspicious lesion of the cavum.

Crystal-storing histiocytosis is a rare condition that was firstly described by Glaus in 1917 [1]. It is histologically characterized by intracellular cytoplasmic crystalline inclusions within histiocytes, which accumulate and affect different organs of the human body (Figure 1) [2,3,4,5]. These inclusions are made of monoclonal immunoglobulin deposits and rarely of polyclonal immunoglobulin deposits or drug material [1,2,3,4,5,6,7,8,9,10,11,12,13,14,15,16,17,18,19,20,21,22,23,24,25,26,27,28,29,30,31,32,33,34,35,36,37,38,39,40,41,42,43,44,45,46,47,48,49,50,51,52,53,54,55,56,57,58,59,60,61,62,63,64,65,66,67,68,69,70,71,72,73,74,75,76,77,78,79,80,81,82,83,84,85,86,87,88,89,90,91,92,93,94,95,96,97,98,99,100,101,102,103,104,105,106,107,108,109,110,111,112,113,114,115,116,117,118,119,120,121,122,123,124,125,126,127,128,129,130,131,132,133,134,135,136,137,138,139,140,141,142,143,144,145,146,147]. A synthesis of the reported cases is presented herein. For more details for every single case reported and the corresponding reference information, the supplementary tables can be consulted (Supplementary Tables S1 and S2). Historically, the initial reports referred to crystal-storing histiocytes as Gaucher cells, as they do resemble Gaucher cells in morphology as well as by special stains. Conversely to this impression, further evaluation with immunohistochemical, immune fluorescence, and electron microscopy studies revealed that this accumulated cytoplasmic eosinophilic material within histiocytes consists of immunoglobulins mostly of monoclonal origin. Therefore, the histiocytes started to be referred to as pseudo-Gaucher cells, like cells in chronic myelogenous leukemia. Furthermore, some authors suggested the term of pseudo-pseudo-Gaucher cells to distinguish them from the already described pseudo-Gaucher cells in chronic myelogenous leukemia and acute leukemia. 

Crystal-storing histiocytosis can be localized and affect one organ or multiple and affect several organs. It is commonly associated with lymphoproliferative conditions especially those with plasmacytic differentiation, including multiple myeloma, lymphoplasmacytic lymphoma, marginal zone lymphoma with plasmacytic differentiation, and monoclonal gammopathy of undetermined significance. It can be less commonly associated with non-neoplastic conditions including autoimmune, infection, inflammatory, and drug-induced etiologies. The prognosis of this lesion is variable and relies on the underlying clinical disease. In this review article, we aim to describe and discuss most of the clinicopathologic characteristics of crystal-storing histiocytosis to increase awareness among pathologists and treating physicians about its important clinical associations and differential diagnoses.

## 2. Clinical Features

The clinical findings based on published cases (Supplementary Table S1) are summarized in Table 1. The two genders are almost equally affected, with a very slight male predominance (50.8%). The age of distribution ranges from 18 years to 91 years at presentation. The clinical presentation in 24.31% (44/181) of the cases is that of an incidental finding or of a notable mass or swelling on clinical examination, imaging study, or screening procedures. Other manifestations vary widely according to the organ involved, including, but not limited to, gastrointestinal, respiratory, bone, renal, neurological, and ophthalmic manifestations. Since the most commonly associated clinical condition is multiple myeloma, the majority of crystal-storing histiocytosis patients also present with multiple myeloma symptoms, such as bone pain and fractures, hypercalcemia, anemia, and renal dysfunction. The presented gastrointestinal manifestations are abdominal pain, diarrhea, melena, weight loss, gastroesophageal reflux disease symptoms, rectal bleeding, a polyp on screening colonoscopy, hepatosplenomegaly, and discoloration of the affected organ in clofazimine-induced crystal-storing histiocytosis. A nonproductive cough is the main indication of lung lesions, with a small percentage of patients presenting with an asymptomatic mass. The renal symptoms are mostly related to renal failure presentation with elevated creatinine levels. The neurological presentation is secondary to brain involvement including memory and speaking problems, muscle weakness, tremors, numbness, transient visual blurring, hemiparesis, progressive vertigo, headaches, diplopia, and altered mental status with intermittent episodes of confusion, disorientation, difficulties with concentration, seizure, and, rarely, mass lesion. The symptoms related to eye involvement are mostly swelling or an induced tumor mass. Other ocular manifestations are conjunctivitis, corneal opacity, diplopia, ptosis, proptosis, external ophthalmoplegia, and a decrease in visual acuity. The lymph nodes are primarily enlarged upon presentation due to primary lymphoma and its accompanied crystal-storing histiocytosis. It was found that the lymph nodes appear with a blackish discoloration in the context of clofazimine use. In breast cases, especially during screening mammography, crystal-storing histiocytosis can be suspicious for a cancer-like mass. 

Typically, no organ is protected from crystal-storing histiocytosis. However, the most affected organs are bone marrow, kidneys, lungs, lymph nodes, skin, and eyes. The stomach is the most involved organ in the gastrointestinal tract, followed by the colon, liver, small intestine, esophagus, and pancreas. The spleen is involved in 3.33% of the cases. In the nervous system, the brain is the most affected, and it is extremely rare that peripheral nerves and ganglions are affected. In the head and neck region, the mucosa is commonly affected, followed by the tongue and salivary glands. The heart and pleura are equally touched by this disease. Rare involvements have been reported for breast, thymus, bone, adrenal gland, urinary bladder, testes, thyroid, retroperitoneum, omentum, pleural fluid, trachea, subcutaneous tissue, adipose tissue, soft tissue, peritoneum, mesentery, teeth, and vessels.

The definitive diagnosis of crystal-storing histiocytosis is made by histological examinations using biopsy specimens and, rarely, resections and cytology samples. Most of the resection specimens involve the lung due to a strong suspicion of lung cancer; otherwise, small biopsies are enough to confirm the diagnosis. Despite being less frequent, generalized forms of crystal-storing histiocytosis exist and appear with more aggressive clinical findings secondary to multiple organ involvement at the time of presentation. Upon a histological diagnosis of crystal-storing histiocytosis, an expanded clinical, radiological, and laboratory workup should be conducted. 

The prognosis is dependent on the anatomic distribution of this condition and, most importantly, on the treatment of the underlying associated disease or on the possibility to stop the causative agent, such as with drug-induced crystal-storing histiocytosis. In 34.58% (46/133) of localized lesions, variable responses to their management upon follow-up were found, with complete resolution and, sometimes, regain of organ function, for the surgically resected lesions and symptoms-free condition upon stopping the causative drug, as observed for clofazimine-induced-crystal storing histiocytosis, or partial remission in some medically treated patients. However, 10.53% (14/133) of the patients died from the underlying associated disease or because of complications. Similarly, for the generalized form, 25.93% (14/54) of the patients died due to the underlying disease or as a result of its complications. For 40.74% (22/54) of the patients with generalized disease, the outcome was variable and showed no response or partial and complete response to the treatment. In some patients, the association of crystal-storing histiocytosis with a lymphoproliferative disease such as multiple myeloma has a good prognosis, as the detection of crystal-storing histiocytosis leads to the early identification and treatment of the associated hematological condition.

The treatment of crystal-storing histiocytosis variably relies on many factors, which include the associated clinical condition and its distribution and the presence of either localized or generalized lesions. For example, the majority of the described cases were associated with multiple myeloma; thus, the mainstay of treatment was chemotherapy that could be followed by a bone marrow transplant. Interestingly, in the case of pulmonary crystal-storing histiocytosis secondary to extranodal marginal zone lymphoma, the lesion may be cured by surgical resection without recurrence on follow-up, especially when it presents as a solitary nodule, with no need for a further aggressive treatment by chemotherapy. Sometimes, observation is the only way of management, as occurs in monoclonal gammopathy of undetermined significance when there is no disease progression. *H. pylori*-induced crystal-storing histiocytosis can be treated by antibiotic-based treatment of *H. pylori* gastritis. In clofazimine-induced crystal-storing histiocytosis, stopping the administration of the causative drug can completely resolve the condition. Taken together, the approach to management varies based on the distribution of crystal-storing histiocytosis, as it can be cured by surgical resection, antibiotics, or eliminating the causative agent from localized lesion. Conversely, the generalized crystal-storing histiocytosis generally needs an aggressive treatment, reflecting the aggressive nature of its associated condition. 

## 3. Clinical Associations

Crystal-storing histiocytosis can be an indirect sign of a more aggressive underlying clinical condition (Table 2). It is well known to be associated with many neoplastic diseases (87.9%); thus, a thorough workup is highly recommended in order to reveal any hidden disorder. The vast majority of associated neoplastic conditions are lymphoproliferative hematological malignancies in 97.83% of the cases. These include multiple myeloma, marginal zone lymphoma, lymphoplasmacytic lymphoma, and monoclonal gammopathy of undetermined significance. Multiple myeloma is the disease most commonly associated with crystal-storing histiocytosis, representing 34.06% of the lesions. It is typically the prototype of plasma cell neoplasms affecting primarily the bone marrow in different body locations through neoplastic monoclonal plasma cell infiltration, resulting in monoclonal immunoglobulin overproduction and subsequent deposition in several organs. It can be diagnosed by laboratory tests such as blood or urine electrophoresis, as well as by bone marrow biopsy specimens that add other diagnostic criteria. Marginal zone lymphoma is the main lymphoma associated with crystal-storing histiocytosis, observed in around 25.36% of the cases. It is mainly reported in extranodal sites. including the lung, eyes, stomach, kidneys, breast, thymus, trachea, tonsil, and parotid. In a few cases of extranodal marginal lymphoma, plasmacytic differentiation is noted histologically. Lymphoplasmacytic lymphoma was demonstrated in 14.49% of patients. Waldenström’s macroglobulinemia is present in 4.35% of patients. It is considered a subtype of lymphoplasmacytic lymphoma that is defined by the presence of detectable blood IgM monoclonal gammopathy. Monoclonal gammopathy of undetermined significance is found in 11.59% of crystal-storing histiocytosis cases. It is usually asymptotic and diagnosed incidentally by protein electrophoresis. This lesion does not show multiple myeloma-related manifestations such as renal dysfunction, hypercalcemia, anemia, and bone lesions. It mainly affects the bone marrow, with less than 10% of plasma cells on bone marrow histological examination. It may progress to multiple myeloma, and a regular follow-up of such patients is recommended. Moreover, crystal-storing histiocytosis has rarely been associated with Waldenström macroglobulinemia, diffuse large B cell lymphoma, plasmacytoma, mantle cell lymphoma, follicular lymphoma, systemic mastocytosis, and myelodysplastic syndrome. Curiously, in the two cases of mastocytosis, both with peripheral eosinophilia, the histiocytes were filled up with Charcot–Leyden eosinophilic crystals, resulting in the so-called Charcot–Leyden crystal-associated crystal-storing histiocytosis [18,145]. In one case, the disease affected the colon, with histologic features of eosinophilic colitis. In addition, crystal-storing histiocytosis can accompany some non-neoplastic lesions (12.1%), including autoimmune, infectious, and inflammatory-related situations. More specifically, Sjogren’s syndrome, *H. pylori* gastritis, rheumatoid arthritis, and Crohn’s disease are the most commonly reported non-malignant conditions. The principal autoimmune disease encountered in association with crystal-storing histiocytosis is Sjogren’s syndrome, followed by rheumatoid arthritis. The site of polyclonal immunoglobulin deposition secondary to Sjogren’s syndrome is mainly the lung. *H. pylori* infection can be an etiology of polyclonal immunoglobulin accumulation, as it is a well-known cause of chronic gastritis with dense plasma cell inflammation. Interestingly, rare cases of crystal-storing histiocytosis are attributed to a drug-related etiology, as well as to inhaled environmental foreign particles such as silicon and asbestos, particularly in the pleura and lung. Drug-induced crystal-storing histiocytosis is typically documented as an adverse secondary reaction to clofazimine, an anti-lepromatous leprosy treatment causing the so-called clofazimine-induced enteropathy. Generally, crystal-storing histiocytosis can be classified in various ways, either based on the associated conditions being neoplastic or non-neoplastic or based on the accumulated deposits being immunoglobulin deposits (93.38%) or non-immunoglobulin deposits (6.62%). The non-neoplastic conditions associated with crystal-storing histiocytosis can be further subdivided according to their etiology into inflammatory (15.79%), autoimmune (31.58%), infection (26.32%), and drug-dependent (26.32%). The immunoglobulin deposit-associated subtype encompasses monoclonal deposits (90.55%), namely, of kappa (78.26%) and lambda (19.13%) light chains, and polyclonal deposits of immunoglobulins (9.45%). Finally, the non-immunoglobulin deposits can be sub-classified according to their causative agent into drug-induced (55.56%), environmental foreign particles (22.22%), or Charcot–Leyden crystals (22.22%), associated with crystal-storing histiocytosis. 

## 4. Pathogenesis

The pathogenesis of crystal-storing histiocytosis is primarily attributed to the overproduction of immunoglobulins by monoclonal plasma cells as well as polyclonal ones in non-neoplastic conditions. Therefore, the pathologic mechanism is largely based on abnormal immunoglobulin production as a result of a dense plasma cell infiltration that can be associated with many conditions, such as myeloma, rheumatoid arthritis, and *H. pylori* gastritis. This leads to the improper intralysosomal degradation of these structurally altered and over-accumulated immunoglobulins that are subsequently deposited within the histiocytes. This improper histiocytic enzymatic degradation of immunoglobulins might be related to an altered conformation of the immunoglobulin light chain structure due to amino acid replacement in the region responsible for its hydrophobic properties, producing immunoglobulin proteins with acquired resistance to lysosomal enzymatic degradation [146]. Interestingly, the immunoglobulins crystals are markedly composed of kappa light-chain protein and, rarely, of lambda light chain. Some authors explained this composition by the soluble nature of kappa immunoglobulins within the lysosome, which makes their crystallization and accumulation within the histiocytes easier than for the lambda light chain [9]. Regarding the histiocytic accumulation of non-immunoglobulin deposits, it reflects the fact that any foreign material or particle can induce a macrophagic reaction which leads to engulfing the histiocytes with defective phagocytosis.

## 5. Pathologic Features

**Figure 2:**
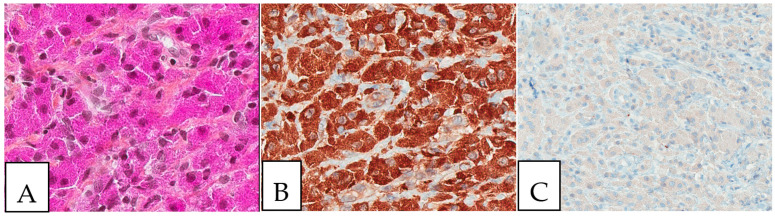
Crystal storing histiocytosis. (**A**) Morphology of samples affected by crystal-storing histiocytosis, (**B**) CD68-positive histiocytes, (**C**) S100-negative lesion. This specimen is from the same patient described in Figure 1 (**A**) and was taken from a colonic white mucosal elevation.

Detailed characteristics are presented in Supplementary Table S2. The size of the lesions ranged from 1 mm to 11 cm. A histopathologic examination is considered the gold standard for crystal-storing histiocytosis, as lesions are constantly composed of sheets of histiocytes that are filled up with crystalline needle-shaped to globular eosinophilic cytoplasmic inclusions (Figure 2). These inclusions are rarely accumulated within nearby plasma cells. In renal crystal-storing histiocytosis, eosinophilic cytoplasmic depositions can be seen in the tubular epithelium and in the glomeruli. This material can be positive or negative on PAS stains and typically negative on Congo red, Masson trichrome, acid-fast bacilli, Grocott–Gomori methenamine silver, Perls, Von Kossa, and Sirius red stains. The cells affected by crystal-storing histiocytosis are uniformly positive for anti-CD68 and anti-CD163 on immunohistochemistry, confirming their histiocytic nature. On the other hand, they are negative for anti-S100, anti-CD1a, anti-smooth muscle actin, anti-CD138, anti-langerin, anti-desmin, and anti-myoglobin. Importantly, due to the high clinical association of this disease with the monoclonal lymphoproliferative process with mostly monoclonal IgM kappa light chain restriction, it seems mandatory to investigate for the presence of lymphoma by asking for a lymphoma immunohistochemistry panel including CD20, CD79a, CD3, CD10, CD5, BCL2, BCL6, cyclin D1, CD23, and immunoglobulins light chains, either by immunohistochemistry or by in situ hybridization, as the first step to determine if it is a monoclonal or a polyclonal process. Typically, the crystals react positively to anti-kappa antibodies and negatively to anti-lambda antibodies in most cases of neoplastic crystal-storing histiocytosis. In contrast, crystals in the presence of non-neoplastic polyclonal crystal-storing histiocytosis will be react positively to both anti-kappa antibodies and anti-lambda antibodies. As the commonest accompanied pathology, multiple myeloma presents histologically with sheets or nodules of neoplastic plasma cells on bone marrow biopsy. There is typically an associated reduction in other hematopoietic cell lineages in cases of diffuse involvement. Interestingly, amyloid deposition secondary to the extracellular accumulation of monoclonal immunoglobulins in multiple myeloma can resemble crystal-storing histiocytosis, as it includes cells with an eosinophilic amorphous morphology. Unlike in crystal-storing histiocytosis, the amyloid disposition is positive for Red Congo stain with apple green Birefringent appearance. In addition, the deposition in crystal-storing histiocytosis is mostly within the histiocytes, whereas the amyloid deposition is typically extracellular, vascular, and perivascular. Immunohistochemically, the neoplastic plasma cells are positive for anti-CD138 and anti-MUM1 and present monoclonal immunoglobulin light chain restriction. Extranodal marginal zone lymphoma is characterized by sheets of small lymphocytes that usually reveal a distinct lymphoepithelial lesion in mucosa-associated lymphoid tissue organs such as the stomach. This lesion demonstrates sometimes a plasmacytic differentiation of the tumor cells. The neoplastic lymphocytes are positive for anti-CD20 and anti-BCL2 and negative for anti-CD3, anti-CD5, anti-CD10, and BCL6.

The autoimmune disease most commonly associated with crystal-storing histiocytosis is Sjogren’s syndrome, which is microscopically characterized, after a biopsy of the affected organs, e.g., the salivary glands, by chronic lymphocytic infiltration with variable degrees of interstitial fibrosis. Moreover, rheumatoid arthritis is another autoimmune disease associated with crystal-storing histiocytosis and can manifest with rheumatoid nodules that can affect many organs, such as the lungs. These nodules show on microscopic evaluation as granulomatous nodular reactions with central necrosis and peripheral histiocytic palisading and chronic inflammation. In the gastrointestinal tract, infectious etiologies are commonly reported in the presence of crystal-storing histiocytosis, especially in the stomach with *H. pylori*-dependent gastritis, which principally affects the antrum and is characterized by plasma cell-rich chronic inflammation with or without activity. Practically, special stains such as the Giemsa stain could be of value in diagnosing *H. pylori* bacilli-shaped bacteria in the stomach or even immunohistochemistry. If left untreated, it may eventually give rise, over time, to more serious conditions such as lymphoma. The deposition material can be rarely secondary to non-immunoglobulin substances, such as in clofazimine-induced crystal-storing histiocytosis, which is characterized by histiocytes with red deposits, and in cytoplasmic Charcot–Leyden crystals within histiocytes that are typically described with mastocytosis and peripheral eosinophilia. On electron microscopy, the histiocytes demonstrate membrane-bound cytoplasmic crystals with variable shapes and sizes. 

## 6. Differential Diagnoses

Due to the morphological similarity of crystal-storing histiocytosis associated with a wide spectrum of conditions with eosinophilic cytoplasmic inclusions, several diseases are included in its differential diagnoses, such as malakoplakia, granular cell tumor, rhabdomyoma, Langerhans cell histiocytosis, Rosai Dorfman disease, Gaucher disease, Russel bodies gastritis, iron pill-induced gastritis or duodenitis, Whipple disease, and amyloidosis (Table 3). Thus, it is necessary to correlate the clinical findings with morphological and immunohistochemical features, in order to reach the correct diagnosis. 

**Figure 3:**
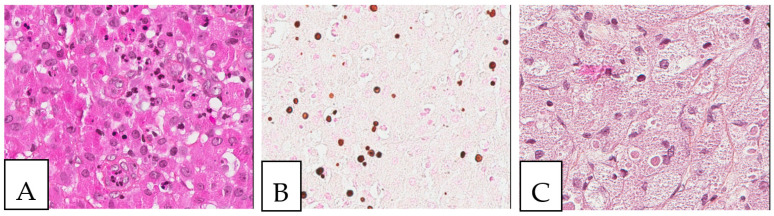
Malakoplakia. (**A**). Malakoplakia is an inflammatory process consisting of macrophages with an abundant eosinophilic cytoplasm (hematoxylin, eosin, Safran). The lesion was found in a 60-year-old male patient who underwent a colonoscopy within the context of pre-renal transplant evaluation and was found to have this lesion as a perianal polyp that was removed by polypectomy. (**B**). The characteristic Michaelis–Gutmann basophilic inclusions are better highlighted by the Von Kossa stain. (**C**). Granular cell tumor with microgranular eosinophilic cytoplasm containing the pustule-ovoid bodies of Milian (eosinophilic globules surrounded by a clear halo). This lesion presented as a reddish skin nodule in a 48-year-old-female patient.

Malakoplakia is an inflammatory condition associated with macrophages that are unable to properly digest the phagocytosed bacterial material. It typically affects the urogenital region, but many organs can be affected. Microscopically, it is characterized by a histiocytic reaction with eosinophilic cytoplasm in the inflammatory background and, sometimes, by Michaelis–Gutmann basophilic inclusions which can be demonstrated by Von Kossa stain (Figure 3). It is positive for anti-CD68 and negative for anti-S100. In contrast, granular cell tumors (Figure 3), a benign tumor type of Schwann cell origin, also with wide anatomic distribution, presents positivity for both anti-CD68 and anti-S100, with homogenous eosinophilic cytoplasm and the presence of the so-called pustule-ovoid bodies of Milian (eosinophilic globules surrounded by a clear halo). It is commonly described in the head and neck and cutaneous tissues. It is sometimes associated with pseudoepitheliomatous hyperplasia of the overlying squamous epithelium that can be mistaken for a squamous cell carcinoma.

**Figure 4:**
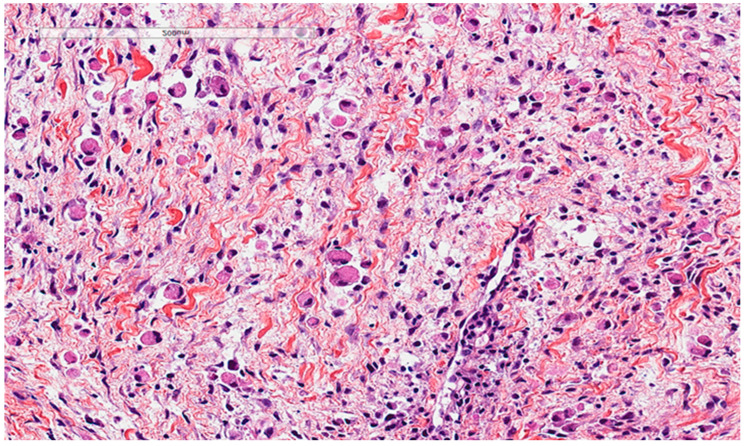
Rhabdomyoma cells also show an abundant eosinophilic cytoplasm. This lesion presented as a vocal cord polyp in a 67-year-old male patient.

Rhabdomyoma is also considered a differential diagnosis. It is a rare benign tumor of skeletal muscle differentiation typically affecting the children and mostly found in the heart and is associated with tuberous sclerosis. However, extracardiac rhabdomyoma does exist, especially in the head and neck region, affecting the adults. It is characterized histologically by clear to eosinophilic polygonal spider cells that are positive for smooth muscle actin, desmin, and myoglobin (Figure 4). 

**Figure 5:**
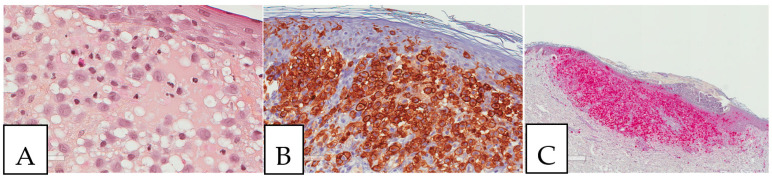
Langerhans cell histiocytosis. (**A**). Cutaneous Langerhans cell histiocytosis with characteristic coffee bean-like cells (**B**). CD1a-positive tumor cells. (**C**). S100-positive tumor cells. The patient was a young female adult presenting with multiple skin lesions, whose biopsy is shown here.

**Figure 6:**
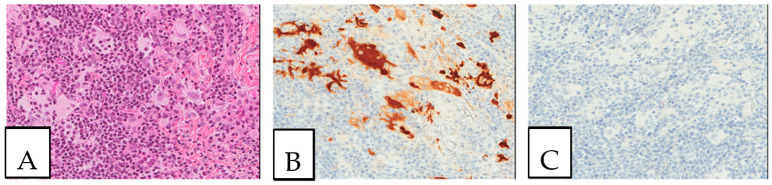
Rosai Dorfman disease. (**A**). Cutaneous Rosai Dorfman disease showing histiocytes with an enlarged eosinophilic cytoplasm with engulfed inflammatory cells (emperipolesis). (**B**). S100-positive lesional cells. (**C**). CD1a-negative lesional cells. This biopsy was performed on a 64-year-old-female patient presenting with pigmented lesions of the back suspicious of regressive melanoma.

Langerhans cell histiocytosis is a multi-system disease of clonal origin, associated with RAS/MAPK pathway mutations, especially the BRAF V600E mutation, which presents morphologically with cleaved coffee bean-like cells in an eosinophil-rich background. The neoplastic cells are positive for anti-S100, CD1a, and langerin (Figure 5). Rosai Dorfman disease is a non-Langerhans cell histiocytosis (Figure 6), usually involving the lymph nodes and the skin, but can also show a disseminated form. It is characterized by histiocytic accumulation with an eosinophilic cytoplasm and, sometimes, cytoplasmic ingested inflammatory cells (emperipolesis). 

**Figure 7:**
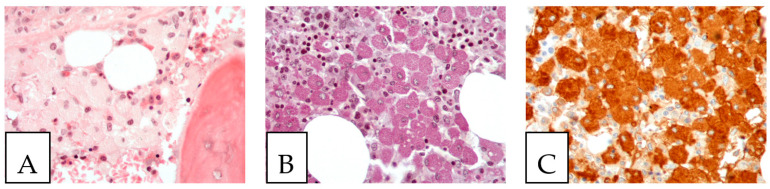
Gaucher disease. (**A**). Bone marrow involvement, with histiocytes with an eosinophilic cytoplasm (Gaucher cells). (**B**). PAS-positive histiocytes. (**C**). CD68-positive histiocytes. The patient, a 52-year-old female, suffered from hepatosplenomegaly and anemia.

**Figure 8:**
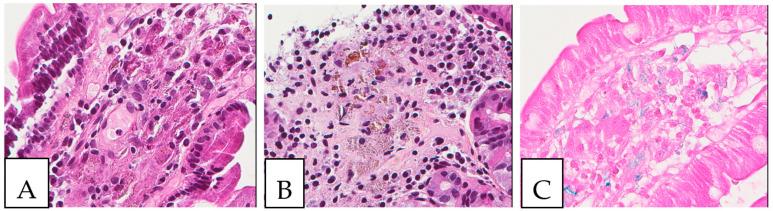
Iron deposits in the gastrointestinal mucosa. Histiocytes with cytoplasmic crystalloid-like pink/brown material in the duodenum (**A**) and the stomach (**B**). (**C**) Positive iron stain (Perl’s) in the duodenum. A 74-year-old male patient with known multiple myeloma presented with diarrhea, and an endoscopy was performed.

**Figure 9:**
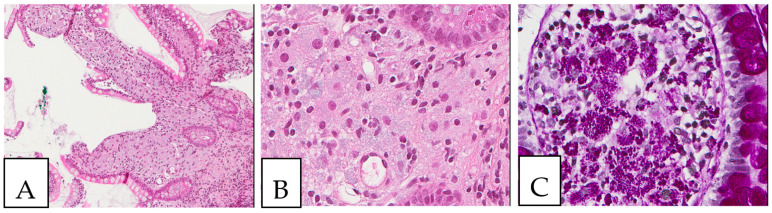
Whipple disease. (**A,B**) Histiocytic accumulation with a foamy to eosinophilic cytoplasm in the duodenal lamina propria. (**C**). PAS-positive histiocytes in the duodenal lamina propria. The biopsy was obtained from a 43-year-old female patient investigated by endoscopy for anemia; PCR was positive for *Tropheryma whipplei*.

**Figure 10:**
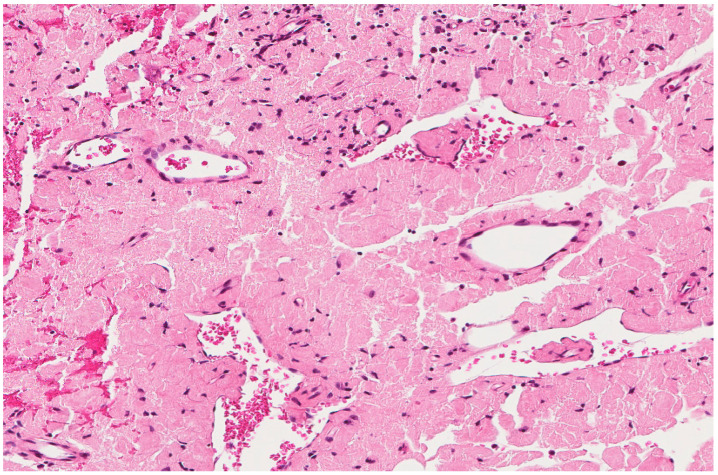
Amyloidosis with eosinophilic amorphous extracellular material. An 80-year-old male with a resected laryngeal polyp.

Gaucher disease, an autosomal recessive lysosomal storage disease, is characterized by the macrophagic accumulation of glucocerebroside affecting many organs, especially the liver, spleen, and bone marrow. It is due to an enzymatic defect, producing histologically the so-called crinkled-paper eosinophilic cytoplasm (Figure 7). In addition, some diseases, such as chronic myelogenous leukemia, can show bone marrow cells with similar morphological findings, called pseudo-Gaucher cells; based on that, some authors described crystal-storing histiocytosis as containing pseudo-pseudo-Gaucher cells (44). In the stomach, Russel body gastritis is considered an important pitfall of gastric crystal-storing histiocytosis, as both can be associated with *H. pylori* gastritis. The principal histological feature of Russel body gastritis is the presence of accumulated polyclonal homogenous round deposits of immunoglobulins within plasma cells cytoplasm, which are positive for anti-CD138. In addition iron pill-induced gastritis or duodenitis can present with histiocytic brown crystalloid-like material with iron-stain positivity (Figure 8). In the duodenum, the histiocytic accumulation in the lamina propria in Whipple disease can be a pitfall of crystal-storing histiocytosis. These histiocytes are consistently PAS-positive (Figure 9). In addition, amyloidosis could make a differential diagnosis of crystal-storing histiocytosis, given that the material is pale pink, glassy, and amorphous; however, it is extracellular, mostly vascular and perivascular, and produces a birefringent apple-green color under polarized light (Figure 10).

Other differentials but rare diagnoses of crystal-storing histiocytosis include mastocytosis, mycobacterium tuberculosis, xanthogranuloma, clofazimine-induced crystal-storing histiocytosis, crystal-storing histiocytosis associated with hereditary cystinosis. Mastocytosis is a neoplastic proliferation of mast cells that can be cutaneous or systemic in presentation. The main histological feature is the presence of neoplastic mast cells infiltration with an eosinophilic cytoplasm in a background rich in eosinophils. It is positive for anti-CD117 and anti-CD25. Mycobacterium tuberculosis is characterized by a caseating necrotizing granuloma in which the organism can rarely be demonstrated by Ziehl Nelson stain; therefore, a PCR test of bacterial DNA is needed for confirmation. Xanthogranuloma is a chronic inflammatory process that shows lipid-laden macrophages with a foamy to eosinophilic cytoplasm. It can be mistaken for a neoplastic process. In addition, patients with leprosy who are on treatment with clofazimine can have gastrointestinal symptoms secondary to clofazimine-induced enteropathy, which is characterized by red drug material within the histiocytes. Finally, hereditary cystinosis is a genetic disease due to a defective cysteine amino acid resulting in its abnormal intra-lysosomal accumulation within the affected cells.

## 7. Conclusions

To conclude, crystal-storing histiocytosis is a rare, probably under-recognized condition that can be the iceberg of a more serious disease; therefore, pathologists and clinicians should be aware of it and perform a detailed workup for any hidden neoplastic lesion. It can be localized or generalized, with a more adverse prognosis in the latter case. The most common associated clinical conditions are lymphoproliferative neoplasms with kappa light chain restriction. The treatment of this condition is largely dependent on targeting the underlying primary cause.

## Figures and Tables

**Table 1:** Clinical findings according to the published cases (see Supplementary Tables).

Characteristics	Case no, (%)
Gender	
Female	89 (49.17%)
Male	92 (50.83%)
Age	
Range	18–91 years
Sample	
Cytology	8 (5.59%)
Biopsy	103 (72.02%)
Resection	32 (22.37%)
Distribution	
Localized	133 (71.12%)
Generalized	54 (28.87%)
Anatomic site	
Bone marrow	43 (15.93%)
Kidney	27 (10%)
Lung	25 (9.26%)
Lymph node	24 (8.89%)
Skin	14 (5.19%)
Eye	13 (4.81%)
Stomach	12 (4.44%)
Colon	11 (4.07%)
Liver	11 (4.07%)
Brain	10 (3.70%)
Spleen	9 (3.33%)
Small intestines	8 (2.96%)
Heart	5 (1.85%)
Pleura	5 (1.85%)
Head and neck mucosa	5 (1.85%)
Esophagus	4 (1.48%)
Breast	4 (1.48%)
Thymus	3 (1.11%)
Pancreas	3 (1.11%)
Tongue	3 (1.11%)
Bone	3 (1.11%)
Salivary gland	3 (1.11%)
Peritoneal fluid	3 (1.11%)
Adrenal gland	2 (0.74%)
Urinary bladder	2 (0.74%)
Testes	2 (0.74%)
Thyroid	2 (0.74%)
Retroperitoneum	2 (0.74%)
Omentum	2 (0.74%)
Pleural fluid	1 (0.37%)
Trachea	1 (0.37%)
Subcutaneous	1 (0.37%)
Adipose tissue	1 (0.37%)
Soft tissue (paraspinal)	1 (0.37%)
Peritoneum	1 (0.37%)
Mesentery	1 (0.37%)
Teeth (periapical)	1 (0.37%)
Vessels	1 (0.37%)
Peripheral nerves and ganglions	1 (0.37%)

**Table 2:** Conditions associated with crystal-storing histiocytosis.

Conditions	Case, *n* (%)
Neoplastic	**138**
*Multiple myeloma*	47 (34.06%)
*Lymphoma*	
- MZL	35 (25.36%)
- LPL	20 (14.49%)
- DLBCL	5 (3.62%)
- MCL	1 (0.72%)
- FL	1 (0.72%)
*MGUS*	16 (11.59%)
*Waldenström macroglobulinemia*	6 (4.35%)
*Plasmocytoma*	4 (2.90%)
*Others*	
- Systemic mastocytosis	2 (1.45%)
- Myelodysplastic syndrome	1 (0.72%)
Non-neoplastic	
*Inflammatory*	**19**
- Crohn disease	1 (5.26%)
- Plasma cell granuloma	1 (5.26%)
- Plasmacytic conjunctivitis	1 (5.26%)
*Auto-immune disease*	
- Sjogren’s syndrome	4 (21.05%)
- Rheumatoid arthritis	2 (10.53%)
*Infection*	
- *Helicobacter pylori*	4 (21.05%)
- *Strongyloides*	1 (5.26%)
*Drug*	
- Clofazimine	4 (21.05%)
- Carbamazepine	1 (5.26%)
	
Immunoglobulin deposits	**127**
*Monoclonal*	115 (90.55%)
- Kappa light chain	90 (78.26%)
- Lambda light chain	22 (19.13%)
*Polyclonal*	12 (9.45%)
	
Non-immunoglobulin deposits	**9**
*Drug*	
- Clofazimine	4 (44.44%)
- Carbamazepine	1 (11.11%)
*Environmental foreign particles*	
- Silicon	1 (11.11%)
- Asbestos	1 (11.11%)
*Charcot–Leyden crystals*	2 (22.22%)

MZL: marginal zone lymphoma, LPL: lymphoplasmacytic lymphoma, DLBCL: diffuse large B cell lymphoma, MCL: mantle cell lymphoma, FL: follicular lymphoma, MGUS: monoclonal gammopathy of undetermined significance.

**Table 3:** Main differential diagnoses of crystal-storing histiocytosis.

Diagnosis	Key Histologic Features	Ancillary Tests
Malakoplakia	Eosinophilic cytoplasm Michaelis–Gutmann inclusions	Von Kossa stain (+ Michaelis–Gutmann inclusions), S100-, CD68+
Granular cell tumor	Homogenous eosinophilic cytoplasm Pustule-ovoid bodies of Milian	PAS, S100+, CD68+
Rhabdomyoma	Spider cells	Desmin+, myoglobin+, SMA+
Langerhans cell histiocytosis	Bean coffee-shaped cells Eosinophil-rich background	S100-, CD1a+, langerin+
Rosai Dorfman disease	Enlarged eosinophilic cytoplasm with emperipolesis	S100+, CD68+, CD1a-, langerin-
Gaucher disease	Crinkled paper cytoplasm	PAS+
Russel bodies gastritis	Round homogenous plasma cells inclusions	CD138+
Iron pill-induced gastritis	Brown crystalloid-like material within histiocytes	Iron stain (Perls)+
Whipple disease	Foamy to eosinophilic histiocytes in duodenal villi	PAS+
Amyloidosis	Eosinophilic amorphous material	Congo red (birefringent apple-green color material under polarized light)

## Data Availability

Not applicable.

## Supplementary Materials

The following supporting information can be downloaded at: https://www.mdpi.com/article/10.3390/diagnostics13020271/s1.
