# Supplementary material for: Crystal-Storing Histiocytosis: The Iceberg of More Serious Conditions
Source: Diagnostics (Basel). 2023 Jan 11;13(2):271. doi: 10.3390/diagnostics13020271 (PMC9858286; doi:10.3390/diagnostics13020271)
Supplement: Supplementary file 1 [file diagnostics-13-00271-s001.zip › diagnostics-2004731-supplementary.pdf]

Crystal-Storing Histiocytosis: The Iceberg of More Serious Conditions

Mousa Mobarki [1,*], Alexandra Papoudou-Bai [2], Jean Marc Dumollard [3], Abdulaziz H. Alhazmi [4], Shaqraa Musawi [5], Mohammed Ali Madkhali [6], Khalid Y. Muqri [7], Michel Péoc'h [3] and Georgia Karpathiou [3]

1 Pathology Department, Faculty of Medicine, Jazan University, Jazan 45142, Saudi Arabia
2 Pathology Department, Faculty of Medicine, University of Ioannina, 47100 Ioannina, Greece;
apapoudou@uoi.gr
3 Pathology Department, University Hospital of Saint-Etienne, 42023 Saint-Etienne, France;
j.marc.dumollard@chu-st-etienne.fr (J.M.D.); michel.peoc'h@chu-st-etienne.fr (M.P.); georgia.karpathiou@chu-st-etienne.fr (G.K.)
4 Microbiology and Parasitology Department, Faculty of Medicine, Jazan University, Jazan 45142, Saudi Arabia; abalhazmi@jazanu.edu.sa
5 Department of Medical Laboratories Technology, College of Applied Medical Sciences, Jazan University, Jazan 45142, Saudi Arabia; smusawi@jazanu.edu.sa
6 Division of Hematology and Oncology, Department of Internal Medicine, Faculty of Medicine, Jazan University, Jazan 45142, Saudi Arabia; mmedkhali@jazanu.edu.sa
7 Faculty of Medicine, Jazan University, Jazan 45142, Saudi Arabia; dr.khalidmuqri@gmail.com
* Correspondence: mamobarki@jazanu.edu.sa; Tel.: +966-540926111

Supplementary Tables

Table S1. Clinical findings based on the literature review

Ref	Location	No	Sex	Age (years)	Associated disease	Clinical presentaion	specimen	Type	F/U
**[1]**	Trachea	1	M	60	Marginal Zone Lymphoma of Mucosa-Associated Lymphoid Tissue	Asymptomatic with solitary tracheal tumor on CT scan	N/A	L	N/A
**[2]**	Lacrimal sac	1	F	middle-aged	Extramedullary plasmacytoma	Painless nodule with epiphora	Biopsy	L	N/A
**[3]**	Case 1: bilateral orbital soft tissues<br>Case 2: lacrimal gland (left)	2	1M<br>1F	62<br>85	Case 1: low grade B-cell lymphoma<br>Case 2: MALT lymphoma	Case 1: right greater than left upper eyelid swelling and epiphora<br>Case 2: incidental finding of an enlarged left lacrimal gland	Biopsy	2L	Case 1: complete resolution<br>Case 2: 50% regression
**[4]**	Bone marrow	1	M	73	Monoclonal kappa light chain gammopathy	Axonal polyneuropathy in all limb	Biopsy	L	N/A
[5]	Orbit and conjunctiva	4	N/A	N/A	1 inflammatory reaction<br>3 localized B-cell lymphomas	2 conjunctival mass<br>2 orbital mass (causing proptosis and hypoglobus)	Biopsy	4L	Retained good vision after treatment
[6]	Bone marrow<br>Lymph node<br>Colon	1	M	69	Bing-Neel syndrome With plasmacytic lymphoma/Waldenstrom macroglobulinemia	Abdominal pain and confusion<br>Lymphadenopathy	Biopsy	G	Clinical improvement after treatment

						hepatosplenomegaly, polyneuropathy.			
[7]	Submandibular skin	1	M	64	Multiple myeloma	Painful swelling of the neck and difficulty swallowing (firm submandibular mass with skin changes)	Biopsy	L	Clinical improvement of the skin lesions decrease in monoclonal protein
[8]	Solitary nodule in the right lower lob (lung)	1	F	64	Sjogren's syndrome	Chronic cough (2years)	Resection	L	No recurrence 6 months after surgery
[9]	Vertebral bone (L1)	1	F	75	MGUS	Acute lower back pain with vertebral fracture	Vertebral bone biopsy	L	Clinical improvement after surgery
[10]	Kidney Bone marrow	1	F	66	Multiple myeloma and renal proximal tubulopathy	Evaluation of kidney dysfunction	Renal and BM biopsies	G	Partial remission of his malignancy
[11]	Stomach	1	M	86	DLBCL	Two episodes of hematemesis and abdominal pain/1 day	Gastric biopsies	L	Regular F/U for DLBCL
[12]	Peritoneal fluid	1	M	72	Multiple myeloma	Evaluation for liver transplantation and refractory ascites	Peritoneal fluid sample	L	Patient passed away after one month of the diagnosis due to complications of his disease
[13]	Kidney	1	F	66	Monoclonal gammopathy of renal significance	Acute kidney injury	Renal biopsy	L	N/A
[14]	Bone marrow	1	F	56	Multiple myeloma	Recurrence of multiple myeloma	Bone marrow core biopsy	L	Very good partial response for chemotherapy and is awaiting autologous stem cell transplantation
[15]	Rectum	1	M	71	Immunoglobulin G kappa monoclonal gammopathy of undetermined significance (MGUS)	Rectal bleeding	Rectal biopsy	L	Alive with no disease progression
[16]	Stomach	1	M	79	MALT lymphoma	Melena and hematemesis (bleeding ulcer)	Gastric biopsy	L	N/A
[17]	Left lower lobe (lung)	1	F	60	Sjogren’s syndrome	Persistent night cough and sputum for months	Wedge resection of left lower lobe	L	No evidence of recurrence after a regular follow-up for three years to date.

[18]	Right colon mass<br>Infiltrating submandibular mass	1	F	75	Indolent IgG-kappa multiple myeloma	Abdominal pain and altered general condition	Colon (right hemicolectomy)<br>Submandibular (biopsy)	G	Death
[19]	Colonic polyp (proximal ascending colon)	1	M	55	Elevated absolute eosinophil<br>count and a positive IgG anti-Strongyloides antibody (parasitic infection)	Screening colonoscopy	Colonic biopsy	L	No relevant clinical issue on F/U
[20]	Renal and retroperitoneal	1	M	57	Lymphoplasmacytic lymphoma	Hypercalcemia following an episode of dizziness	Renal and retroperitoneal biopsy	G	N/A
[21]	Bone marrow	1	F	58	Multiple myeloma amyloid	Back pain, anorexia, nausea, vomiting, and fatigue of 3-week duration	Bone marrow biopsy	L	Complete response to treatment despite persistence of both CSH and amyloid
[22]	Liver<br>Bone marrow	1	M	79	MGUS	Pleural effusion osteolytic lesion of the humerus increased AST and cholestatic markers	Biopsy	G	N/A
[23]	Bone marrow	1	F	72	Plasma cell myeloma<br>Carbamazepine use for peripheral neuropathy	Chronic renal insufficiency, hypothyroidism, peripheral neuropathy, hypercholesterolemia, anxiety, and tremor	Biopsy	L	N/A
[24]	Bone marrow<br>Liver<br>Lymph nodes<br>Spleen<br>Kidney	1	M	65	Myelodysplastic syndrome (MDS) with multilineage dysplasia, extracellular kappa light chains crystalline nephropathy	Vomiting, diarrhea, left flank pain, shivers without fever	Autopsy specimen	G	The patient died from a gastrointestinal bleeding
[25]	Bone marrow<br>Pleura	1	F	73	Past history of IgG kappa monoclonal gammopathy	Asthenia, weight loss and bone pain of the limbs	Biopsy	G	N/A
[26]	Case 1: extra pulmonary para spinal soft tissues<br>Case 2: Stomach<br>Bone marrow	2	1F<br>1M	68<br>49	Case 1: lost to follow up<br>Case 2: multiple myeloma	Case 1: dyspnea, increasing fatigue and a remote history of pneumonia<br>Case 2: progressive weight loss, an inability to eat, burning upper abdominal pain and weakness	Biopsy	Case 1: L<br>Case 2: G	Case 1: lost to follow up<br>Case 2: died
[27]	Kidney	3	M<br>M<br>M	71<br>67<br>74	Case 1: history of B cell lymphoproliferative disorder and Waldenstrom macroglobulinemia<br>Case 2: MGUS, membranoproliferative glomerulonephritis	Case 1: Anephrotic-range proteinuria of 3.6 g/day and serum creatinine of 1.18 mg/dL<br>Case 2: diabetes, hypertension, obesity, and	Biopsy	3L	Case 1: DOD<br>Case 2: stable<br>Case 3: DOD

					Case 3: multiple myeloma and membranoproliferative glomerulonephritis	myelodysplastic syndrome, ANCA-negative pauci-immune glomerulonephritis with arteritis and features of thrombotic microangiopathy<br>Case 3: Acute renal failure			
[28]	Bone marrow	1	M	79	Multiple myeloma<br>End stage renal disease	Bilateral comminuted femur fracture	Biopsy	L	N/A
[29]	Subcutaneous Lymph nodes	1	F	74	Splenic diffuse large B-cell lymphoma (DLBCL), (non-GCB) monoclonal gammopathy	Multiple subcutaneous masses, abdominal pain, and fever	Biopsy	G	Partial remission
[30]	Bone marrow	1	F	83	Multiple myeloma<br>Breast cancer	Severe pancytopenia	Biopsy	L	N/A
[31]	8 Bone marrow<br>1 Eye/orbit,<br>1 Liver and Skin,<br>1 Omentum	8	7M<br>1F	Average age at diagnosis is of 62 years (range, 40-73 years)	4 multiple myelomas<br>2 MGUS<br>1 low grade B cell lymphoma<br>1 systemic amyloidosis with myeloma	Persistent shoulder pain<br>Severe headache and hypertension<br>Elevated total protein<br>Thrombocytopenia, splenomegaly, osteolytic lesion<br>Progressing fatigue<br>Nausea, vomiting and abdominal pain<br>Renal failure and anemia	Biopsy	5 L<br>3 G	5 alive with persistent disease after either chemotherapy or bone marrow transplantation<br>1 died 3 years after PCM diagnosis
[32]	Stomach	4	2M<br>2F	Average age, 69 y; range, 56 to 82 y)	2 extranodal marginal zone lymphomas of MALT type, kappa-restricted<br>1 diffuse large B-cell lymphoma, kappa-restricted<br>1 mantle cell lymphoma, kappa-restricted<br>2 associated with Helicobacter pylori 1 of them showed a lymphocytic gastritis pattern	Gastroesophageal reflux abdominal pain<br>melena diarrhea	Biopsy	4L	Persistent disease on follow up in all patients then 2 died of their disease
[33]	Caudal brain stem and cerebellum (posterior fossa)	1	M	36	Gout, hypertension, hyperlipidemia, traumatic brain injury, peripheral neuropathy of unclear etiology, and obstructive sleep apnea	History of progressive vertigo, headaches, diplopia and altered mental status with intermittent episodes of confusion, disorientation and difficulties with concentration	Biopsy	L	Complete spontaneous resolution of the lesion

[34]	Kidney	1	M	60	IgG kappa light chain multiple myeloma<br>Nephrotic syndrome<br>Renal dysfunction	Progressive bilateral decreased vision	Biopsy	L	Hematologic response improvement of the papilledema and macular edema, the patient developed dialysis-dependent end-stage renal failure
[35]	Thymus	1	F	22	Thymic extranodal marginal zone lymphoma with plasmacytic differentiation	Thymic mass	N/A	L	N/A
[36]	Left neck of femur	1	M	70	Plasma cell myeloma	Left sided pain around the hip with pathological fracture of left neck of femur	Resection	L	Under follow up for his myeloma
[37]	Bone Marrow<br>Kidney	1	M	48	Kappa light chain multiple myeloma<br>Light chain proximal tubulopathy<br>Myeloma cast nephropathy	Uremia and anemia	Biopsy	G	No recovery of renal function after multiple myeloma treatment
[38]	Bilateral multiple lung mass lesions	1	F	38	MALT lymphoma	Asymptomatic (health screening)	Biopsy	L	N/A
[39]	Kidney	1	M	67	had a monoclonal gammopathy of renal significance<br>(IgG kappa monoclonal gammopathy)<br>Membranoproliferative glomerulonephritis	Rise in creatinine to 1.8 mg/dl from a baseline of 1.3 mg/dl, proteinuria, hematuria, new rash, ascites, and leukopenia	Biopsy	L	Progressive disease with renal and hepatic failure and myocardial infarction with ileus
[40]	Cervical lymph nodes	1	M	70	History of squamous cell carcinoma of the right base of the tongue	Enlarged cervical lymph node	Biopsy	L	Disease free after 10 months
[41]	Bone marrow<br>Lymph node<br>Stomach<br>Esophagus<br>Breast<br>Skin<br>Upper back<br>Inner cheek<br>Duodenum<br>Colon<br>Spleen	13	7M<br>6F	60 years (range, 33-79)	3 plasma cell myelomas<br>3 lymphoplasmacytic lymphomas<br>7 Extranodal marginal zone B-cell lymphomas	Persistent progressive abdominal pain<br>Fatigue<br>Rib pain<br>Anemia<br>Lymphadenopathy<br>Cyanosis<br>Stomach cramps, rectal bleeding; stomach nodules on EGD<br>Abnormal mammogram<br>Skin lesion<br>Inner cheek mass<br>Lymphocytosis		10 L<br>3 G	5 alive<br>4 died<br>4 unknown
[42]	Small	1	F	68	Diffuse large B-cell (double hit) lymphoma	Postprandial abdominal pain,	Biopsy	L	Resolving of symptoms after

	bowel and terminal ileum				Disseminated M. abscessus infection of the skin treated by clofazimine and tigecycline	diarrhea, weight loss, and melena with Endoscopic evaluation revealed black pigmentation of the duodenum			stopping of clofazimine
[43]	Kidney	1	M	68	Low-grade B-cell lymphoproliferative disorder (marginal zone Lymphoma)	Kidney failure and accompanying fatigue, myalgia, arthralgias, and lower-extremity edema	Biopsy	L	N/A
[44]	Skin	2	1F<br>1M	80<br>52	Case 1: Waldenström macroglobulinemia<br>Case 2: multiple myeloma	Case 1: bilateral periorbital oedema with yellow discolouration<br>Case 2: pruritic rash on his back during chemotherapy	Biopsy	2L	Case 1: indolent clinical course but died 6 years later from transformation into a diffuse large B cell lymphoma in the bone marrow<br>Case 2: died 4 years later after a protracted clinical course involving multiple cycles of chemotherapy and numerous complications
[45]	Breast	1	F	62	Extranodal marginal zone lymphoma (MZL)	Breast mass on screening mammography	Biopsy	L	N/A
[46]	Bone marrow	1	F	57	Plasma cell myeloma	IgA κ monoclonal proteinemia and monoclonal proteinuria	Biopsy	L	N/A
[47]	Conjunctiva	1	M	58	Extranodal marginal zone B-cell lymphoma (EMZL)	Tumour of the right (OD) eye of 2 years duration	Biopsy	L	Disease free after 5 years
[48]	Intra cerebral	1	M	56	No associated disease (No previous history of lymphoproliferative disease)	Intra-cerebral tumor-like lesion	Biopsy	L	N/A
[49]	Lymph nodes<br>Kidney<br>Spleen<br>Bone marrow	1	M	69	A defined lymphoplasmacytic disease could not be demonstrated	Mesenteric lymph node enlargement and proteinuria, including a monoclonal k LC	Biopsy	G	Clinical and biological improvement after treatment

[50]	Lung<br>Lymph nodes<br>Bone marrow	1	F	53	Lymphoplasmacytic lymphoma<br>Waldenstrom macroglobulinemia	Fatigue, nonproductive cough, dyspnea, night sweats and recurrent pyelonephritis	Biopsy	G	Clinical improvement with treatment
[51]	Posterior neck<br>Right breast<br>Left upper arm	1	F	91	Marginal-zone lymphoma with plasmacytic differentiation	Enlarging mass	Biopsy	G	No significant change on follow up
[52]	Left renal hilum	1	M	77	Marginal zone B-cell lymphoma	Slow growing mass	Biopsy	L	Refused treatment and lost on follow up
[53]	Bone marrow	1	F	43	MGUS	Stage IV chronic kidney disease	Aspirate & biopsy	L	N/A
[54]	Right orbit	1	M	53	MALT lymphoma	8-year history of a slowly enlarging tumor	N/A	L	N/A
[55]	Right breast	1	F	30	No past history of LP-PCD	Non palpable breast mass	Biopsy	L	Unremarkable
[56]	Waldeyer's ring (tonsils)	1	F	71	Marginal zone B-cell lymphoma<br>of mucosa-associated lymphoid tissue (MALT-lymphoma)	Swelling of the bilateral cheeks and tonsils	Biopsy	L	Cheeks swelling decreased with rituximab therapy
[57]	Right parietal lobe	1	F	38	IgA restricted plasma cell dyscrasia	Tonic-clonic seizure	Biopsy	L	Stable brain MRI on follow up
[58]	Stomach (greater curvature)	1	N/A	N/A	Occult lymphoproliferative disorders	Longstanding symptoms suggestive of gastroesophageal acid reflux despite proton pump inhibitor use	Biopsy	L	N/A
[59]	Lung	1	F	54	Marginal zone lymphoma of mucosa associated lymphoid tissue	Asymptomatic solitary lung nodule	Right upper and middle bilobectomy	L	N/A
[60]	Stomach (greater curvature)	1	F	55	Sjogren's syndrome<br>H. pylori gastritis	Asymptomatic<br>Endoscopy: flat, whitish, finely granular membrane	Endoscopic submucosal dissection	L	No hematologic disease or recurrence of gastric lesions developed
[61]	Left temporal lobe	1	F	20	No associated disease	Progressive worsening headaches with prior sore throat	Biopsy	L	Neurollogically intact
[62]	Right eye (conjunctiva)	1	M	32	Ocular extranodal marginal zone lymphoma	Conjunctival tumour of the right eye	Biopsy	L	Conservative treatment after complete resection of the tumor

[63]	Lung (upper left lobe)	1	M	80	No underlying LP-PCD	N/A	Wedge resection	L	Disease free without any treatment
[64]	Bone marrow	1	F	78	Monoclonal gammopathy of undetermined significance (MGUS)	Asymptomatic	Bone marrow aspirate and biopsy	L	N/A
[65]	Eye<br>Kidney<br>Bone marrow	1	F	62	MGUS<br>Fanconi syndrome	Decrease in visual acuity<br>Asthenia<br>Weight loss	Biopsy	G	Complete hematological ophthalmological, and renal remission
[66]	Left sided maxillary sinus extending to the hard palate	1	F	72	Monoclonal plasma cell proliferation<br>Plasmacytoid dendritic cell tumour<br>Stable chronic myelomonocytic leukaemia	Enlarged left cervical lymph node followed slowly growing ulcerated left palatal lesion left-sided maxillary sinus lesion	Biopsy	L	Stable
[67]	4 lung<br>1 pleura	5	3 F<br>2 M	F/54<br>F/89<br>F/50<br>M/63<br>M/68	2 B-cell marginal-zone lymphomas<br>2 monoclonal gammopathy of undetermined significance<br>1 pulmonary plasmacytoma	3 symptomatic<br>2 incidental finding	1 pulmonary lobectomy<br>3 wedge resections<br>1 pleural biopsies	5L	3 alive<br>2 DOD
[68]	Duodenum<br>Jejunum	1	M	19	Multibacillary leprosy<br>Clofazimine treatment	Abdominal pain	Biopsy	L	Improved upon stopping clofazimine
[69]	Upper lip and cheek	1	F	51	Mild lymphoplasmacytic infiltrate exhibiting IgM lambda<br>light chain restriction	Submucosal swelling of the left upper lip and cheek of 2 weeks duration	Excision	L	Asymptomatic with no treatment
[70]	Brain	1	F	32	Plasma cell malignancy	Transient visual blurring, right hemiparesis, and spells of aphasia	Biopsy	L	Good response to treatment
[71]	Maxillary left lateral incisor	1	M	38	No associated hematologic disorder or systemic disease	Persistent periapical	Excision	L	Patient lost on follow up
[72]	Case 1: lung<br>Case 2: skin (right forearm)	2	1M<br>1F	64<br>57	Case 1: extranodal MZL with kappa light chain restriction<br>associated with more prominent plasma cell differentiation<br>Adenocarcinoma<br>Case 2: extranodal MZL with surface kappa light chain<br>restriction	Case 1: bilateral lung masses<br>Case 2: soft tissue mass	Case 1: FNA, biopsy<br>Case 2: FNA biopsy	2L	N/A
[73]	Bone marrow<br>Liver<br>Spleen	1	F	48	MGUS	Hepatosplenomegaly for 2 yr	Bone marrow aspirate and biopsy and liver specimen	G	Persistent clinical improvement on follow-up

[74]	Brain	1	F	27	Crohn disease	Left-sided tremor and rnumbness and a tingly sensation	Biopsy	L	N/A
[75]	Tongue (midline of the dorsum)	1	M	67	Plasmacytoma	Lump on the posterior dorsum of the tongue	Biopsy	L	Clinical improvemen t
[76]	Ascetic fluid	1	F	50	Waldenström disease	Recurrent ascites Abdominal pain	Fluid	L	N/A
[77]	Left breast	1	F	54	Marginal zone B cell lymphoma of mucosa-associated lymphoid tissue with plasmacytic differentiation	Breast focal densities identified on screening mammography	FNA and biopsy	L	N/A
[78]	Bone marrow	1	M	63	Multiple myeloma	Progressive weakness<br>Weight loss<br>Bone pains	Biopsy	L	N/A
[79]	Bone marrow	1	N/A	N/A	N/A	N/A	Bone marrow aspirate	L	N/A
[80]	Pleura	1	M	68	Pulmonary silicosis<br>Monoclonal gammopathy of undetermined significance	Asymptomatic	Pleural biopsy	L	Alive on follow up
[81]	Kidney<br>Bone marrow	3	M<br>M<br>M	52<br>70<br>65	Case 1: Fanconi syndrome<br>Case 2: large B-cell lymphoma , Fanconi syndrome<br>Case 3: multiple myeloma	Case 1: acute renal failure<br>Case 2: chronic renal failure<br>Case 3: acute worsening of chronic renal failure	Biopsy<br>Smear	3G	Case 1: patient died of sepsis<br>Case 2: patient died of sepsis<br>Case 3: on haemodialys is
[82]	Lung (right lower lobe)	1	F	75	Monoclonal gammopathy of undetermined significance (MGUS)	Chronic nonproductive cough	FNA biopsy	L	No size progression of the nodule on follow up
[83]	Bone marrow	1	F	91	Aggressive systemic mastocytosis	Cough shortness of breath and seizures	Biopsy	L	N/A
[84]	Thymus	1	F	53	Sjögren's syndrome<br>Thymic MALT lymphoma	Incidental CT findings of mediastinal tumor	Resection	L	No recurrence after 15 months of follow up
[85]	Heart (right atrium)	1	M	64	No lymphoma	Supraventricular tachyarrhythmia and a right atrial mass	Resection	L	No current follow up information
[86]	Eye	1	M	66	Bilateral crystalline keratopathy<br>Monoclonal gammopathy of undetermined significance	Unilateral progressive ptosis, proptosis, and external ophthalmoplegia	Biopsy	L	N/A
[87]	Bone marrow<br>Kidney	1	M	66	Multiple myeloma	Finding of monoclonal serum proteins during the work-up of	Biopsy	G	Partial remission upon treatment

						acute renal insufficiency			
[88]	Lung (right middle lobe)	1	M	64	Lung asbestos exposure	Chronic pulmonary consolidation	Biopsy	L	Patient refused surgical treatment and continued on symptomatic treatment
[89]	Lymph node	1	F	76	Nodal marginal zone lymphoma	Axillary lymphadenomegaly	Biopsy<br>Excision specimen	L	Followed in 26 months with another, but genetically unrelated, lymphoma with the same IgL chain usage, however, without induction of CSH
[90]	Bone marrow	1	M	54	Multiple myeloma	Loose motions for 2 years	Biopsy	L	Died of his disease
[91]	Kidney<br>Bone marrow	1	M	65	Plasma cell clonality	Renal transplant	Biopsy	G	N/A
[92]	Brain (bilateral white matter)	1	F	31	Atypical plasma cell infiltrate suspicious for plasma cell proliferative disorder	Recurrent spells of aphasia and right-sided weakness for 2 years	Biopsy	L	Slight improvement in imaging findings 17 months post-biopsy
[93]	Brain (right frontal extra-axial)	1	F	72	Meningioma<br>No history of hematological disease	1-year history of memory disturbance	Resection	L	Symptoms free 6 months post follow up
[94]	Ocular adnexa (left lower eyelid)	1	F	81	MALT lymphoma with plasmacytic differentiation	5-month history of a slowly enlarging tumor	Excision specimen	L	No recurrence for 1 year after surgery
[95]	Stomach (the angle, anterior wall of the antrum, distal antrum along the greater curvature)	1	F	56	Polyclonal plasma cell proliferation caused by Helicobacter pylori gastritis	Dyspepsia and epigastric pain for 2 weeks<br>Endoscopy: 3 Polyps and chronic gastritis	Polypectomies	L	N/A
[96]	Right and transverse colon	1	F	78	Past medical history of dermatologic mastocytosis and peripheral eosinophilia	Diarrhea and weight loss<br>Colonoscopy: polyposis	Subtotal colectomy	L	N/A
[97]	Lung	1	M	52	Multiple myeloma	Chronic bilateral pulmonary infiltration	Biopsy	L	N/A
[98]	Skin (anterior aspect of the chest and on the neck)	1	F	62	Multiple myeloma IgG Kappa	Large red infiltrated verrucous lesion	Biopsy	L	Died of his illness

[99]	Case 1:<br>Bone and bone marrow, liver, spleen<br>Case 2:<br>Bone and bone marrow, liver, peritoneal fluid (ascites)	2	1M<br>1F	49<br>72	Case: MGUS<br>Case 2: IgA kappa-type multiple myeloma	Case 1: fatigue and weight loss<br>Case 2: diagnosed with ascites after a 3-year long history of asthenia, anorexia, weight loss, sweats and fever, epigastric pain, and deep bone pain in both legs	Biopsy	2G	Case 1: DOD<br>Case 2: alive 6 years after diagnosis
[100]	Kidney	1	M	41	Multiple myeloma IgD k-monoclonal<br>Light chain cast nephropathy (LCCN), k type.<br>Light chain Fanconi's syndrome (LCFS), k type	Worsening neck pain and weight loss	Biopsy	L	Partial improvemen t
[101]	Lung (left lower lobe)	1	F	69	MALT lymphoma	Asymptomatic lung mass on routine chest x ray	Resection	L	Good 4 months after follow up
[102]	Pleural fluid	1	F	79	IgA κ multiple myeloma	Right thoracic pain	Pleural fluid (cytology)	L	DOD 1 month after diagnosis
[103]	Kidney	1	F	70	No history of hematological malignancy	Progressive renal insufficiency	Biopsy	L	Patient died about 6 months later of a stroke related to her peripheral vascular disease
[104]	Kidney	1	M	45	Multiple myeloma IgG k monoclonal	Elevated creatinine	Biopsy	L	Alive
[105]	Lung (left upper lobe)	1	F	50	Long history of rheumatoid fac-tor–negative rheumatoid arthritis<br>No associated hematologic disorder	Lung mass	Wedge resection	L	N/A
[106]	Bone marrow<br>Kidney	1	F	62	Multiple myeloma	Anemia<br>Acute renal failure	Biopsy	G	Good response to the treatment
[107]	Lymph nodes<br>Omentum	1	F	44	Lepromatous leprosy with history of adverse reaction to clofazimine	Blackish discoloration of lymph node, omentum and peritoneum<br>Rectosigmoid adenocarcinoma	Biopsy	L	Symptomati cally well
[108]	Bone marrow<br>Lungs<br>Kidney<br>Liver	1	M	51	Multiple myeloma	6-month history of progressive fatigue, episodic fevers, back pains without	Biopsy	G	DOD

						radiological lesions, slight anemia and minimal hepatosplenomegaly			
[109]	Bone marrow<br>GI tract	1	F	74	Multiple myeloma	Epigastric pain, vomiting, poor appetite, weight loss and pain in the left side of the chest	Biopsy	G	DOD
[110]	Right middle lobe	1	M	59	Extranodal marginal-zone B-cell lymphoma	Right lower chest pain and right upper abdominal complaints and weight loss	Lobectomy	L	No recurrence
[111]	Kidney	1	M	58	Marginal zone lymphoma	Acute renal failure	Biopsy	L	N/A
[112]	Bone Marrow<br>Liver<br>Skin<br>lymph nodes<br>Peritoneum<br>Pericardium<br>Pleura<br>Retroperitoneum<br>Lungs<br>Myocardium<br>Kidneys<br>Adrenals<br>Testes<br>Mucosa of the gastrointestinal tract	1	M	73	MGUS	IgA kappa paraproteinemia and paraproteinuria	Biopsy	G	DOD
[113]	Left eye (lacrimal gland)	1	F	62	Orbital extranodal marginal zone B-cell lymphoma (EMZL)	Diplopia and swelling of the palpebral conjunctiva of the left eye	Biopsy	L	N/A
[114]	Lymph node	1	F	53	Follicular lymphoma	Left neck mass	Biopsy	L	No recurrence
[115]	Bone marrow<br>Spleen	1	M	54	Lymphoplasmacytic lymphoma (LPL)	N/A	Biopsy<br>Resection	G	No complete response
[116]	Small intestines<br>LN	1	M	32	Clofazimine treatment for leprosy	Chronic abdominal<br>Pain<br>Red skin discoloration	Biopsy	G	DOD
[117]	LN (left cervical)	1	F	77	DLBCL	Fever<br>Weight loss<br>Diffuse adenopathy	Biopsy	L	No complete remission
[118]	Bone marrow<br>Lymph node	12	7 M<br>5 F	Mean age at	5 Multiple myeloma	**MM patients:**<br>Bone pain	Biopsy<br>Resection	11 G<br>1 L	**MM patients:**

	Stomach<br>Lung<br>Thymus<br>Skull<br>Spleen			diagnosis of 59 years (range, 35 to 77 years	6 Lymphoplasmacytic lymphomas (one in thymus associated with lupus)<br>1 plasma cell granuloma	Elevated serum proteins<br>Hypercalcemia<br>Anemia<br>Acute renal Failure<br>**LPL patients:**<br>Splenomegaly<br>Symptoms of hyperviscosity<br>Renal failure<br>Ig G or IgM serum paraproteinemia<br>**Plasma cell granuloma patient:**<br>Solitary lung nodule			4 with persistent CSH after treatment<br>1 DOD<br>**LPL patients:**<br>5 with persistent CSH after treatment<br>1 transformation to DLBCL with persistent CSH<br>1 with persistent disease after treatment<br>**Plasma cell granuloma patient:**<br>No recurrence on last follow up
[119]	Right lung	1	F	72	Lymphoplasmacytoid lymphoma	Incidental	N/A	N/A	N/A
[120]	Bone marrow<br>Spleen<br>Lymph node<br>GI tract<br>Liver<br>Heart pericardium and myocardium)<br>Kidney (interstitium)<br>lungs (interstitium and alveolar spaces)<br>Pancreas<br>Pleura<br>Mesentery	1	M	44	IgG-kappa type lymphoplasmacytic lymphoma (immunocytoma)	Clinical picture of multifocal fibrosclerosis (with mesenteric panniculitis, peritoneal, mediastinal and orbital fibrosis)	Biopsy<br>Resection on autopsy	G	DOD
[121]	Tongue	1	F	73	Rheumatoid arthritis and polyclonal hypergammaglobulinemia	Solitary mass in the tongue	N/A	L	N/A
[122]	Left parotid gland<br>Left peri parotid LN	1	F	81	MALT lymphoma with plasmacytic differentiation	History of long-standing left parotid gland enlargement	FNA<br>Biopsy	L	Complete remission
[123]	Skin (left axilla)	1	M	64	LPL	Clinical diagnosis of Weber-Christian disease	Biopsy	L	Free of symptoms after treatment

[124]	Skin (trunk , left upper arm, breasts)<br>LN	1	F	61	LPL	Multiple tumors	Excision Biopsy	G	N/A
[125]	Lung	1	F	54	Reactive	Asymptomatic solitary lung mass	N/A	L	N/A
[126]	Neck (left posterior cervical)<br>Right parotid	1	M	49	LPL	Left posterior cervical mass	N/A	L	Recurrence in right parotid gland, 18 months later as aggressive B-cell lymphoma
[127]	Case 1:<br>Lymph node<br>Otolaryngic mucosa<br>Bone marrow<br>Kidney<br>Case 2:<br>Cervical lymph node<br>Case 3 :<br>Right lateral wall of the nasopharynx	3	3 F	18<br>77<br>78	LPL	Case 1: bilateral parotid area enlargement<br>Case 2: right neck mass<br>Case 3: mass in right lateral wall of the nasopharynx	Biopsy	2 G<br>I L	Case 1: DOD<br>Case 2: free of disease 8 years later<br>1 lost on follow up
[128]	Cornea<br>Kidney<br>Choroid plexus	1	M	75	5-year history of IgG-kappa-type multiple myeloma	Corneal opacity and chronic renal failure	N/A	G	N/A
[129]	Bone marrow<br>Liver<br>Spleen<br>Skin<br>Lymph node<br>Kidney<br>Intestines<br>Lung<br>Urinary bladder<br>Myocardium<br>Pancreas<br>Thyroid<br>Vessels	1	M	60	IgA kappa MM<br>Amyloidosis	Nocturnal bilateral thighs pain<br>Anemia<br>Loss of appetite<br>Constipation	Biopsy<br>Resection on autopsy	G	DOD
[130]	Left palatal tonsil	1	F	51	Extramedullary plasmacytoma	Slight difficulty in swallowing<br>Mass in the left palatal tonsil	Biopsy followed by resection	L	No recurrence
[131]	Supra clavicular LN	1	M	63	LPL	Anemia	Biopsy	L	Stable after treatment
[132]	Renal tubular cells<br>Leydig's cells<br>Adrenocortica l cell	1	M	52	MM	N/A	N/A	G	N/A

[133]	Conjunctiva	1	F	17	Plasmacytic conjunctivitis	Bilateral papillary conjunctivitis	Biopsy	L	N/A
[134]	Left inguinal Lymph node	1	M	62	Plasmacytoma	Painless swelling in the left groin Subcutaneous swelling, in the left inguinal region	Resection	L	No recurrence after removal
[135]	Bone marrow	1	F	55	MM	Back, chest, hip and flank pain	Biopsy	L	DOD
[136]	Bone marrow<br>Lymph node<br>Liver<br>Spleen<br>Kidney<br>Pancreas<br>GI tact<br>Adipose tissues<br>Adrenal glands<br>Testes<br>Lung<br>Heart<br>Tongue<br>Salivary glands<br>Thyroid<br>Urinary bladder<br>Peripheral nerves and ganglions<br>dura mater<br>Cerebrum	1	M	58	No MM (MGUS)	Bence Jones proteinuria, K type light chain proteinemia and hypogammaglobu linemia	Autopsy	G	DOD
[137]	Left eye (conjunctiva and cornea)<br>Bone marrow	1	M	50	Dysproteinemia (probably of the multiple myeloma type)	Anemia and renal failure	Biopsy	G	N/A
[138]	N/A	N/A	N/A	N/A	N/A	N/A	N/A	N/A	N/A

F/U: follow up, M: male, F: Female, N/A: not available, L: localized, G: generalized, MALT: mucosa associated lymphoid tissue, DLBCL: diffuse large B cell lymphoma, MGUS: monoclonal gammopathy of uncertain significance, LPL: lymphoplasmacytic lymphoma, DOD: died of disease, MM: multiple myeloma

Table S2. Histopathologic findings of literature review

Ref	Cases, n	Size (mm)	H&E	Special stains (PAS Masson's trichrome)	IHC	Ig type	EM
[1]	1		CSH	N/A	CD68 (+)	Ig kappa light chain (+) on IHC	N/A
[2]	1		CSH	N/A	N/A	N/A	N/A
[3]	2	Case 1: 25 x 26 x 14 mm & 19 x 21 x 7 mm Case 2: 20 x 9 x 25 mm	CSH (Linear crystals Rhomboidal crystals)	N/A	CD68 (+)	Ig kappa light chain (+) on IHC	N/A
[4]	1	N/A	CSH (globular)	N/A	CD68 (+)	Ig kappa light chain (+) on IHC	N/A
[5]	4	N/A	N/A	N/A	N/A	N/A	N/A
[6]	1	N/A	Rare histiocytes with crystalline cytoplasmic inclusions were identified (BM smears, LN imprints and colonic biopsy)	N/A	N/A	Immunoglobulin-M lambda monoclonal protein	N/A
[7]	1	N/A	Numerous histiocytes with abundant cytoplasm containing eosinophilic crystals	N/A	N/A	Immunoglobulin G k-restricted plasma cells positive for kappa light chains	Crystals within the cytoplasm of the histiocytes
[8]	1	12 × 10 mm	Histiocytes with abundant eosinophilic cytoplasm	N/A	CD68 (+), S-100 (−),	Kappa (+), lambda (+), immunoglobulin (Ig) G (+), IgM (weak+), and IgA (−) Polyclonal	N/A
[9]	1	N/A	Histiocytes with intracellular amorphous material	N/A	CD68 (+)	Kappa light chain (+)	Abundant bright, amorphous material, and some tubular structures

[10]	1	N/A	Histiocytes was filled with eosinophilic crystalline material	N/A	CD68 (+)	Kappa monoclonal plasma cells (+)	Intracytoplasmic electron dense, polygonal-, rhomboid- (most frequent), or needle-shaped crystalline structures
[11]	1	N/A	Histiocytes with abundant eosinophilic cytoplasm and the presence of linear crystals	N/A	CD68 (+) PancCK, LCA, CD20 (-)	Strongly lambda positive with weak kappa staining	N/A
[12]	1	N/A	Cells with abundant foamy and granular cytoplasm	N/A	CD68 (+) CD138 (-)	N/A	N/A
[13]	1	N/A	Crystal storing histiocytes	N/A	N/A	No specific staining of κ chain, and λ chain on tubular epithelial cells, interstitium, and vessels	Prominent electron-dense crystalline inclusions
[14]	1	N/A	Crystal storing histiocytes	PAS (faint staining) Trichrome (bright red staining)	CD68 (+)	kappa light chain restriction of plasma cells on IHC and ISH	N/A
[15]	1	20 mm × 10 mm	Diffuse sheets of eosinophilic histiocytes with Linear, non-polarizable cytoplasmic crystal-like striations	N/A	N/A	N/A	N/A
[16]	1	10 mm	Lamina propria with prominent collections of large eosinophilic mononuclear cells containing fibrillary crystalloid inclusions	N/A	CD68 (+) lambda light chain desmin, smooth muscle actin, S100 protein, CD117, CD1a, and cytokeratin (all negative)	kappa light chain-restricted immunoglobulin crystals	N/A
[17]	1	15 × 11 mm	Aggregates and sheets of epithelioid macrophages with brightly eosinophilic cytoplasmic crystal materials	Congo red (-)	CD68(+) CD163(+) S-100(-) CD1a(-)	Clonal restriction of the κ-light chain	N/A

[18]	1	N/A	Numerous histiocytes with intracytoplasmic eosinophilic crystals	N/A	CD163(+)	Anti-kappa antibody (+) anti-lambda antibody(-)	N/A
[19]	1	N/A	Crystal-storing histiocytes containing Charcot–Leyden crystals	N/A	N/A	Non immunoglobulin crystals	N/A
[20]	1	N/A	Histiocytes with abundant eosinophilic cytoplasm	N/A	CD68(+)	Kappa light chain (+) on ISH and IHC	Cytoplasmic crystals were identified within the podocytes, glomerular endothelial cells, mesangial cells, proximal tubule cells and interstitial histiocytes
[21]	1	N/A	Sheets of large histiocytic cells with abundant foamy and granular cytoplasm	Congo Red (positive staining inside the cytoplasm of the histiocytic cells)	CD68(+)	No light chain restriction	Focal aggregates of fibrillary amyloid deposits.
[22]	1	N/A	Eosinophilic crystals in cytoplasm of both histiocytes and hepatocytes	Rod-shaped eosinophilic crystals in cytoplasm of both histiocytes and hepatocytes	NA	Kappa light chain (+)	Crystals in the hepatocyte cytoplasms
[23]	1	N/A	Cells filled with eosinophilic crystalloid material	N/A	CD68 (+)<br>CD163 (+)	Kappa light chain	N/A
[24]	1	N/A	Diffuse infiltration of histiocytes with large cytoplasm and intracytoplasmic crystalloid inclusions (BM,LN, liver, spleen)<br>Numerous large needle-shaped or rhomboid-like crystals (renal tubules)	Congo red (-)<br>Renal tubules crystals stain strongly yellow with the Masson's trichrome	CD68 (+)	Kappa and lambda light chains (-)	Abundant intracytoplasmic electron dense rhomboid-like or hexagonal-like crystals within histiocytes of the liver, spleen, BM, and lymph nodes. Crystals of kidney tubular lumen were also massive and were electron dense, without periodic organization of the substructure
[25]	1	N/A	Crystal inclusion in the cytoplasm of histiocytes	N/A	CD68 (+)<br>S100 (-)<br>CD1a (-)	Kappa light chain (+)	N/A

[26]	2	Case 1: 47<br>Case 2: N/A	Histiocytes with needle to rhomboid like cytoplasmic crystals	N/A	CD68 (+)	Kappa light chain (+)	N/A
[27]	3	N/A	Case 1: Histiocytes like cells with eosinophilic cytoplasmic material<br>Case 2: capillary loops with histiocytes like cells having eosinophilic cytoplasmic inclusion<br>Case 3: Histiocytes like cells with cytoplasmic eosinophilic material	Case 1: trichrome (+) bright fuchsinophilic cytoplasmic crystals)<br>Case 2: double contours of the capillary walls on silver stain<br>Case 3: Case 1: trichrome (+) bright fuchsinophilic cytoplasmic crystals)<br>PAS (+)	Case 1: CD68 (+)<br>Case 2 : CD68 (+) CD163 (+)<br>Case 3: CD68 (+)	Case 1: IgM kappa light chain (+ on IHC and IF)<br>Case 2: Kappa IgG light chain on IF<br>Case 3: Kappa IgG light chain (+ on IHC and IF)	Case 1: Crystal laden macrophages with hexagonal crystal of varying sizes<br>Case 2: Kappa gold particles labeling spherical crystals<br>Case 3: capillary loops with several oval electron dense crystal within its cytoplasm
[28]	1	N/A	Sheets of histiocytes with abundant eosinophilic cytoplasm	N/A	CD68 (+)<br>CD163 (+)<br>COX2 (+)<br>pSTAT3 (+)	Kappa light chain (+)	Intracytoplasmic diamond and rhomboid crystals within histiocytes
[29]	1	55 × 3 × 15	Large polygonal and spindle histiocytes eosinophilic cytoplasm with needle shaped crystals	Congo red (-)	CD68 (+)<br>CD163 (+)<br>IgM heavy chain (+)<br>Igκ light chain (+ weak)<br>NB: associated with IgH and Igκ rearrangements	IgM kappa (+)	N/A
[30]	1	N/A	Increased number of histiocytes with crystalline cytoplasmic inclusions	N/A	CD68 (+)<br>CD163 (+)	Kappa light chain (+)	N/A
[31]	8	N/A	Histiocytes with intra cytoplasmic needle like to globular eosinophilic crystal	N/A	CD68 (+)<br>CD163 (+)	Kappa light chain (+) in all cases	N/A

[32]	4	N/A	Full-thickness expansion of the lamina propria by a lymphohistiocytic infiltrate and macrophages with abundant eosinophilic cytoplasm containing nonrefractile, nonpolarizable fibrillary cytoplasmic inclusions	GMS (-) PAS/D (-) AFB (-)	CD68 (+) CD163 (+)	Kappa and lambda light chains (-) in macrophages	Macrophage cytoplasmic inclusions were electron-dense, polygonal-shaped structures
[33]	1	28 × 23 X 19	Gaucher like cells clusters or individually characterized by oval bland oval bland nuclei and abundant eosinophilic cytoplasm with layered PAS-negative intracytoplasmic fibrillary material	Layered PAS-negative intracytoplasmic fibrillary material	CD68 (+)	Kappa light chain (+) on IHC and ISH	Accumulation of elongated osmiophilic and needle-like crystalloid inclusions located in the cytoplasm of numerous histiocytes
[34]	1	N/A	Intra cytoplasmic crystals	Intracytoplasmic fuchsinophilic crystals on trichrome	N/A	Intra cytoplasmic crystals positive for anti-kappa on immunogold staining	N/A
[35]	1	N/A	Numerous histiocytes with abundant cytoplasm filled with crystalline and/or globular eosinophilic material	N/A	CD68 (+)	Lambda light chain (+)	N/A
[36]	1	N/A	Sheets of histiocytes rich in needle shaped intracytoplasmic crystals	PASD (-) Masson Trichrome (bright stain)	CD68 (+)	Kappa light chain (+)	N/A
[37]	1	N/A	Eosinophilic cuboid- and rhomboid-shaped crystals in the cytoplasm of proximal tubular epithelial cells	N/A	N/A	Kappa light chain (+) on IHC	Electro-dense cuboid-, rhomboid-, or needle-shaped crystalline inclusions in proximal tubular epithelial cells and interstitial histiocytes
[38]	1	N/A	Dense proliferation of large, spindle-shaped to rounded cells with abundant, deeply eosinophilic cytoplasm and a single round to ovoid nucleus	Multiple intracytoplasmic crystals, not only in the histiocytes, but also in the plasma cells, but not in the extracellular space	CD68 (+)	Kappa light chain (+) on IHC	Multiple intracytoplasmic crystals, in the histiocytes, and plasma cells

[39]	1	N/A	Numerous crystals in the glomerular capillary lumen and along the capillary wall	Masson Trichrome (+)<br>Silver methenamine (+)	CD68 (+)	Kappa light chain (+)<br>on IHC and IF	Crystals within macrophages as recognized by numerous cytoplasmic vacuoles
[40]	1	N/A	Numerous eosinophilic round crystalline structures admixed with histiocytic nuclei surrounded by fibrosis and an infiltrate of histiocytes and plasma cells	PAS (+)<br>Congo red (+)<br>Von Kossa (-)<br>GMS (-)<br>AFB (-)	CD68 (+)<br>CD163 (+)	No	Amyloid fibril formation (beta-pleated amyloidal fibers)
[41]	13	N/A	Histiocytes with numerous crystalline inclusions within the cytoplasm	PAS (+)	CD68 (+)<br>CD163 (+)	5 Kappa light chain (+)<br>4 Lambda light chain (+)	N/A
[42]	1	N/A	Intestinal mucosa with focal villous blunting and widening due to numerous crystal-laden macrophages infiltrating the superficial lamina propria, consistent with crystal-storing histiocytosis	N/A	N/A	N/A	N/A
[43]	1	N/A	Glomeruli showed prominent infiltration of the capillary loops by large vacuolated cells that contained pink granular material in the cytoplasm	Congo red (-)	CD68 (+)	Lambda light chains (+ on IF & IHC)	Numerous crystals within histiocytes
[44]	2	N/A	Dermal infiltration by crystal storing macrophages	Case 1:<br>PAS (-)<br>Case 2:<br>PAS (-)<br>Congo red (-)	CD68 (+)	Case 1: Kappa light chain & IgM heavy chain (+) on IHC<br>Case 2: Ig light chain (-)	N/A
[45]	1	N/A	Sheets of histiocytes with bright eosinophilic cytoplasmic crystals	N/A	CD163 (+)	Lambda light chains (+ on IHC)	N/A
[46]	1	N/A	Plasma cells & histiocytes with numerous crystalline intracytoplasmic inclusions	N/A	CD68 (+)	Kappa light chain (+) on IHC	N/A

[47]	1	N/A	Histiocytes sheets of with abundant eosinophilic cytoplasm	N/A	CD68 (+)	IgM Kappa light chain (+) on IHC	N/A
[48]	1	N/A	Large collections of pleomorphic cells with intracytoplasmic fibrillar and crystalloid inclusions	N/A	CD68 (+)	No	N/A
[49]	1	N/A	Large sheets of macrophages filled with crystalline needle-like inclusions	Toluidine blue (+) Congo red (-) Masson trichrome (Fuchsinophilic crystals within cytoplasms of tubular epithelial cells)	CD68 (+)	Kappa light chain (+) on IHC	Needle-like crystals within the cytoplasm of macrophages
[50]	1	N/A	Crystal-storing histiocytic infiltrate	N/A	CD68 (+)	IgM lambda light chains (+ on IHC)	N/A
[51]	1	N/A	Diffuse infiltrate of large histiocytic cells with abundant eosinophilic laminated crystals	N/A	CD68 (+)	IgM kappa light chain (+ on IHC)	N/A
[52]	1	41 to 70	Histiocytes filled with eosinophilic refractile crystals	N/A	CD68 (+)	Kappa light chain (+ on IHC)	N/A
[53]	1	N/A	Numerous aggregated histiocytes with intra-cytoplasmic eosinophilic inclusions	N/A	N/A	Elevated serum IgD k monoclonal protein on serum protein electrophoresis and immunofixation study	N/A
[54]	1	N/A	CSH	N/A	CD68 (+)	N/A	N/A
[55]	1	12 x 9 x 7	Histiocytes with abundant eosinophilic granular to palisaded inclusions in the cytoplasm	N/A	CD68 (+)	Predominant lambda expression in plasma cells (on ISH)	Rectangular to rhomboid shaped crystalline structures

[56]	1	N/A	Diffuse infiltration of eosinophilic histiocytes	PAS (-)<br>GMS (-)	CD68 (+)	IgG and kappa light chain (+ on IHC)	N/A
[57]	1	N/A	Histiocytes with needle-shaped eosinophilic cytoplasmic inclusions	N/A	CD68 (+)	IgA/kappa light chain (+ on IHC, ISH and mass spectrometry)	N/A
[58]	1	20	Histiocytes filled with abundant polarisable, crystalline material	N/A	N/A	Kappa-restricted plasma cells on ISH	N/A
[59]	1	19	Histiocytes with y abundant eosinophilic cytoplasmic inclusions	N/A	CD68 (+)	Kappa light chain (+ on IHC and ISH)	N/A
[60]	1	10	Infiltration of abundant histiocytes including acidophilic crystalline structured material	N/A	CD 68 (+)<br>CD163 (+)	Kappa & lambda light (+) on IHC	N/A
[61]	1	35 × 13 × 19	Numerous histiocytes contain rhomboid and needle-like	PAS (+)	CD68 (+)	Kappa & lambda light (+) on IHC<br>Lambda light (+) on IF	Histiocytes with intracellular rhomboid and needle-like inclusions
[62]	1	N/A	Proliferation of histiocytes with eosinophilic crystals	N/A	CD68 (+)	N/A	Rhomboid crystals within the cytoplasm of histiocytes
[63]	1	19 x 29 x 52	Histiocytes with eosinophilic crystals	N/A	CD68 (+)	Kappa (weakly +)<br>Lambda (-)	N/A
[64]	1	N/A	Numerous globular crystalline-laden macrophages	N/A	N/A	N/A	N/A

[65]	1	N/A	Numerous crystal-containing macrophages	Congo red on renal biopsy (-)	CD68 (+)	Negative on IF	Cytoplasmic needle-shaped crystals altered epithelial cells and macrophages
[66]	1	N/A	CSH	N/A	N/A	Kappa light (+) on IHC	N/A
[67]	5	N/A	Histiocytes with intracytoplasmic needle shaped crystals	N/A	CD68 (+)	Kappa light (+) on IHC	N/A
[68]	1	N/A	CSH	N/A	N/A	N/A	N/A
[69]	1	15	CSH	PAS (-)	CD68 (+)	Lambda light (+)<br>Kappa (weak +)<br>on IHC	Elongated and rhomboid shaped<br>dense crystals within the cytoplasm of histiocytes
[70]	1	N/A	CSH	N/A	N/A	Kappa light chain (+) on IHC and ISH	N/A
[71]	1	10	CSH	PAS (-)<br>AFB (-)	CD68 (+)	IgG, kappa and lambda (+ weak)	Histiocytes with cytoplasmic prolongations
[72]	2	N/A	Histiocytes with pale cytoplasmic<br>rod-like and crystalline shapes and crystals on Romanowsky stained smears	AFB (-)	N/A	N/A	N/A
[73]	1	N/A	CSH	N/A	CD68(+)	Kappa light chain (+) on IHC	N/A

[74]	1	N/A	CSH	N/A	N/A	N/A	Needle like inclusions irregular-to-rhomboid-shaped in cross-section within histiocyte cytoplasm
[75]	1	8	CSH	PAS (-)<br>Sirius red (-)	CD68 (+)	IgG (+) on IHC<br>Kappa light chain (+) on ISH	Globular crystalline structures
[76]	1	N/A	Macrophages with needle-like crystals	N/A	N/A	N/A	N/A
[77]	1	28 x 19	Histiocytes with refractile eosinophilic cytoplasmic crystalline	N/A	N/A	Kappa light chain (+) on IHC	N/A
[78]	1	N/A	Numerous globular and crystalline-laden histiocytes	N/A	CD68 (+)	N/A	N/A
[79]	1	N/A	CSH	N/A	N/A	Kappa light chain (+) on IHC	N/A
[80]	1	N/A	CSH	N/A	CD68 (+)	Kappa light chain (+) on IHC	N/A
[81]	3	N/A	CSH	Congo red (-)	CD68 (+)	Kappa light chain (+) on IHC	Needle-shaped and oval crystals in proximal tubular cells
[82]	1	11	Macrophages with needle shaped cytoplasmic crystals	N/A	N/A	N/A	N/A

[83]	1	N/A	Macrophages containing eosinophilic crystalline inclusions	N/A	CD68 (+)	N/A	Macrophages with intra cytoplasmic rod-like or hexagonal inclusions
[84]	1	80 x 30 x 20	Macrophages with globular eosinophilic cytoplasmic inclusion	PAS (weak +) Congo red (-)	CD68 (+) CD163 (+)	IgG-kappa light chain (+)	N/A
[85]	1	3 fragments 15 x 14 x.3 , 11 x 6 x 4 ,43 x 39 x 11	Histiocytes with needle shaped eosinophilic cytoplasmic inclusions	PAS (-) Congo red (-) Von Kossa (-) AFB (-)	CD68 (+)	Polyclonal weak immunoreactivity for IgA, IgG, kappa and lambda light chains on IHC and ISH	Numerous sticklike, trapezoidal, or polygonal osmiophilic crystals and lysosomes in the cytoplasm of histiocytes
[86]	1	N/A	CSH	Congo red (-)	CD68 (+)	Lambda Ig light chain (+) on IHC	N/A
[87]	1	N/A	CSH	Congo red (-)	CD68 (+)	N/A	Variable shaped crystals in tubular lumina and histiocytes
[88]	1	45 x 26 x 40	Sheets of histiocytes with needle like or rhomboidal-shaped eosinophilic cytoplasmic crystals	Congo red (-)	CD68 (+)	(+) IgG and IgA heavy chains and kappa and lambda light chains IgM heavy chain (weak focal +)	Electron dense rhomboid and needle-shaped Crystals within histiocytes
[89]	1	N/A	CSH	PAS (-)	CD68 (+)	Lambda Ig light chain (+) on IHC	Histiocytes with crystalline material
[90]	1	N/A	CSH	PAS (-) Perls (-)	CD68 (+)	N/A	N/A
[91]	1	N/A	CSH	N/A	N/A	N/A	Variable rhomboid to spindle crystal forms

[92]	1	N/A	CSH	N/A	CD68 (+)	Kappa light chain (+) on ISH	N/A
[93]	1	46 x 54	CSH	N/A	CD68 (+)	Kappa and lambda light chain (+) on IHC	Needle or rectangular shape crystal
[94]	1	20	CSH	N/A	CD68 (+)	IgM-kappa light chain (+) on IHC	Histiocytes with cytoplasmic rectangular or rod shaped crystalloids
[95]	1	30 mm, 15 mm and 20 mm	Histiocytes with intracytoplasmic eosinophilic needle shaped crystals	Giemsa (+ H. pylori)	CD68 (+)	Kappa and lambda light chain (+) on IHC<br>IgG and IgA (+) on IF	Variable shapes crystals (amorphous, needle or rhomboid)
[96]	1	numerous polyps ranging from 1 to 7 mm	Histiocytes with eosinophilic cytoplasmic crystals and surrounded by intense eosinophilic infiltrate	N/A	CD68 (+)	N/A	Intracytoplasmic material identical to Charcot-Leyden crystals within histiocytes, bipyramidal, hexagonal, and irregular electron-dense crystals within macrophages
[97]	1	N/A	Histiocytes with eosinophilic cytoplasmic globular inclusion	PAS (+)<br>Congo red (-)	N/A	Kappa light chain (+) on IHC	Amorphous substances without a crystalline shape or fine ultrastructure of lattice or linear parallel configuration indicating storage of noncrystallized immunoglobulin
[98]	1	N/A	CSH	Masson Trichrome (+)	CD68 (+)	N/A	N/A
[99]	2	N/A	CSH	N/A	CD68 (+)	Kappa light chain (+) on IHC	Case 2: geometrical rectangular or cylindrical in configuration cytoplasmic crystals
[100]	1	N/A	Histiocytes and tubular epithelial cells with eosinophilic cytoplasmic crystal shaped inclusions	PAS (weak to -)<br>Trichrome (polychromatic)<br>Congo red (-)	CD68 (+)	Kappa light chain (+) on IHC	Tubular epithelium with electron dense rod-shaped and rhomboid-shaped intracytoplasmic crystalline

[101]	1	20 x 17 x 20	CSH	N/A	CD68 (+)	N/A	Numerous rhomboidal and needle-shaped crystals within the cytoplasm of both histiocytes and plasma cells
[102]	1	N/A	CSH	PAS (-)<br>Congo red (-)	CD68 (+)	IgA heavy and κ light (+) chains on IHC	N/A
[103]	1	N/A	CSH	PAS (+)	N/A	N/A	Inclusions with internal crystalline substructure
[104]	1	N/A	CSH	N/A	N/A	N/A	N/A
[105]	1	20 x 15	CSH	PAS (-)<br>Trichrome (-)<br>AFB (-)	CD68 (+)	Kappa and lambda light chain (+)<br>IgM heavy chain (+)	Intralysosomal deposition of polygon-shaped amorphous crystals, characteristic of immunoglobulin in the histiocytic cells
[106]	1	N/A	CSH	N/A	N/A	N/A	CSH
[107]	1	N/A	Crystal accumulation within sinus histiocytes	PAS (+)<br>AFB (weak +)	N/A	N/A	N/A
[108]	1	25 mm on lobectomy specimen	CSH	PAS (+)	CD68 (+)	Kappa light chain and IgG (+)	Rhomboid electron-dense particles
[109]	1	N/A	CSH	N/A	CD68 (+)	Lambda light chain (+) on IHC	N/A

[110]	1	8 mm and 20 mm	CSH	N/A	CD68 (+)	Polyclonal kappa and lambda light chain	Numerous intracytoplasmic electron dense crystalline bodies within histiocytes
[111]	1	N/A	CSH in the mesanguim	N/A	CD68 (+)	Lambda light chain (+) on IF	Histiocytes with needle-shaped and rhomboid crystals
[112]	1	N/A	Histiocytes with with needlelike crystalline material	Elastica-van Gieson,<br>Giemsa<br>Perls<br>PAS<br>Congo red	CD68 (+)	IgA and<br>IgG heavy chains (+)<br>Kappa and lambda light chains (+)	Variable electron-dense crystalline inclusions with Rectangular and rhomboid shapes
[113]	1	10 mm	CSH	PAS (-)<br>Congo red (-)<br>Trichrome (red)	CD68 (+)	Kappa light chain (+)<br>Heavy chain IgM (+)	N/A
[114]	1	60 x 45	CS neoplastic cells not in the histiocytes	PAS (-)	CD68 (-)	Lambda light chain (+) on IHC, ISH	Rectangular & rhomboidal crystals
[115]	1	N/A	CSH	N/A	N/A	N/A	N/A
[116]	1	N/A	Histiocytes with Red clofazimine crystals	PAS (-)<br>AFB (-)	N/A	All negative	Clear crystal of variable size with osmiophilic bodies
[117]	1	N/A	CSH	N/A	CD68 (-)	Lambda light chain (+) on IHC	N/A
[118]	12	N/A	Crystal inclusions in plasma cells and histiocytes	N/A	CD68 (+)	**MM patients**:<br>5 Kappa light chain<br>3 IgA<br>1 IgM<br>1 no heavy chain<br>**LPL patients:**<br>5 IgM<br>4 kappa light chain<br>2 lambda light chain (one associated with lupus has also polyclonal aspects)<br>**Plasma cell granuloma:**	Rhomboidal and needle-shaped crystals in plasma cell granuloma patients

						polyclonal	
[119]	1	N/A	Sheets of globoid and spindle-shaped crystal-storing histiocytes	N/A	N/A	Anti-kappa immunoglobulin Ig M (+)	Membrane bound
[120]	1	N/A	CSH	PAS (+)<br>Congo red (-)	CD68 (+)	IgG-kappa light chain (+) on IHC	Histiocytes with crystalline inclusions of variable sizes and shapes, (rectangular or needle shaped)
[121]	1	N/A	CSH	N/A	N/A	Polyclonal	N/A
[122]	1	100 x 50 mm	CSH	N/A	CD68 (+)	IgM heavy-chain (+)<br>Lambda light-chain (+)	Polygonal or rectangular membrane bound crystalline inclusions of varying lengths and sizes stored in the cytoplasm of the tumor cells
[123]	1	N/A	CSH	PAS (+)<br>Elasfica-Masson stain red)<br>Iron stain (-)<br>Fat stain (-)<br>Ziehl-Neelsen (-)	CD68 (+)	IgG lambda light chain (+) with a<br>small number of IgM-positive cells	Variable size and shape membrane bounded crystal inclusions
[124]	1	5 mm to 80 mm	CSH	N/A	CD68(+)	IgM, IgG, Ig kappa and Ig lambda (+)	variable shapes membrane bound rhomboid or rectangular to ovoid crystals
[125]	1	N/A	CSH	N/A	N/A	polyclonal	N/A
[126]	1	N/A	CSH	N/A	CD68(+)	B-cell kappa monoclonality on gene rearrangement studies	Elongated and rhomboid, membrane-bound, cytoplasmic crystals

[127]	3	Case 3: 20 mm x 15 mm<br>Case 1 & 2: N/A	CSH	N/A	CD68 (+)	Neoplastic cells in case 1 & 2 : IgM kappa light chain (+) on IHC<br>And IgA kappa light chain (+) on IHC<br>CSH in case 2 : IgM kappa light chain (+) on IHC	Membrane bound rhomboidal crystals
[128]	1	N/A	CSH	N/A	N/A	kappa light and gamma heavy chains on IF	Inclusions were filled with fine crystalline hexagonal columns
[129]	1	Multiples nodule with size range of 10 mm to 110 mm	CSH	PAS (-)<br>Congo red (-)<br>Trichrome (red)	N/A	IgA kappa light chain (+)	Crystal inclusions with variable shapes and size
[130]	1	40 x 40x 20 mm	Crystal deposits in histiocytes and plasma cells	PAS (+ or -)<br>Congo red (+ or -)	N/A	Kappa & lambda light chains (+) on IHC but IgG-kappa was found by immunoelectrophoresis	Crystal inclusions with variable shapes and size (from rhomboidal to hexagonal to polygonal)
[131]	1	N/A	Macrophages with granular eosinophilic cytoplasm	PAS (+)	N/A	IgM Kappa light chain (+) on IHC	Phagocytic cells with electron dense granules
[132]	1	N/A	Crystal inclusions in plasma cells and macrophages But not in plasma cells	N/A	N/A	N/A	N/A
[133]	1	N/A	Crystal inclusions in macrophages and plasma cells	PAS (+)<br>Trichrome (red brown)	Muramidase (+)	IgG on IHC	Membrane bounded crystals
[134]	1	60 x 49 x 55 mm	Crystal inclusions in macrophages and plasma cells	PAS (+)	Muramidase (+)	IgG kappa light chain (+) on IHC	Needle-like crystals
[135]	1	N/A	Crystal inclusions in macrophages and plasma cells	PAS (+)	N/A	Kappa light chain (+) on IF	Crystal inclusions of variable size and shape

[136]	1	N/A	Crystatal inclusions in plasma cells and macrophages	PAS (+/-)<br>Trichrome (red)<br>Congo red (-)	N/A	Ig kappa light chain (+) on IHC	Crystal inclusions with variable shapes and size
[137]	1	N/A	Needle crystal inclusions in macrophages and plasma cells	PAS (-)	N/A	N/A	Variable size and shape crystal deposits
[138]	N/A	N/A	N/A	N/A	N/A	N/A	N/A

H&E: hematoxylin and eosin stain, IHC: immunohistochemistry, Ig: immunoglobulin, EM: electron microscopy, CSH: crystal storing histiocytosis, N/A: not available, κ: kappa light chain, λ: lambda light chain, PAS: periodic acid Schiff stain, ISH: in situ hybridization, AFB: acid fast bacilli

References

1. Kimura S, Oyama Y, Ziyao W, Waseda R, Nishino N, Sakata T, et al. A Rare Case of Tracheal Crystal-Storing Histiocytosis Associated with Marginal Zone Lymphoma of Mucosa-Associated Lymphoid Tissue. Int J Surg Pathol. 2022;

2. Lee CM, Asilnejad B, Cohen LM, Roelofs KA, Rootman DB, Khanlou N, et al. Solitary Extramedullary Plasmacytoma of the Lacrimal Sac with Associated Crystal-Storing Histiocytosis. Vol. 38, Ophthalmic Plastic and Reconstructive Surgery. Ophthalmic Plast Reconstr Surg; 2022. p. 102–7.

3. Eslami M, Rossman D, Rasmussen S, Chae T. Localized ocular crystal-storing histiocytosis and associated lymphoma - Report of two cases and review of literature. Am J Ophthalmol Case Reports. 2022 Mar;25.

4. Lesesve J, Thomas B. Crystal-storing histiocytosis associated with monoclonal kappa light chain gammopathy. Int J Lab Hematol. 2022 Apr;

5. Wilde C, Amin S, Poitelea C, Uddin J, Rose GE, Sherafat H. Orbital Crystal-Storing Histiocytosis: A Clinicopathologic Study of 4 Cases. Ophthal Plast Reconstr Surg. 2021;E89–91.

6. Lequain H, Gerfaud-Valentin M, Fontaine J, Ferrant E, Grumet P, Jamilloux Y, et al. Crystal-storing histiocytosis and Bing-Neel-like syndrome revealing a small B-cell lymphoma with plasmacytic differentiation, presumed to be a marginal zone lymphoma. Clin Case Reports. 2021 Dec;9(12).

7. de Haart SJ, Leguit RJ. Crystal-storing histiocytosis associated with smoldering myeloma. Blood. 2021;138(4):354.

8. Kiya S, Morino S, Iwasaki K, Nakamura A. Surgical resection of pulmonary crystal-storing histiocytosis with Sjögren's syndrome: A case report. Int J Surg Case Rep. 2021 Aug;85.

9. Wiese-Hansen H, Leh F, Hemsing AL, Reikvam H. Immunoglobulin-storing histiocytosis: A case based systemic review. Vol. 10, Journal of Clinical Medicine. J Clin Med; 2021.

10. Ungari M, Ghiringhelli P, Marchi G, Fisogni S, Lavazza A, Molteni A, et al. Combined renal proximal tubulopathy and crystal storing histiocytosis in a patient with κ lightchain multiple myeloma. Pathologica. 2021 Aug;113(4):285–93.

11. Bansal N, Puri P, Nagpal N, Naithani R, Gupta R. Lambda-Restricted Crystal-Storing Histiocytosis of Stomach: A Case Report and Review of Literature. Cureus. 2021 May;13(5).

12. Hamadeh F, Smith MR, Hsi ED. Crystal-storing histiocytes in ascites leading to a diagnosis of plasma cell myeloma. Diagn Cytopathol. 2021 Sep;49(9):E336–9.

13. Nakamura Y, Kitamura H, Ikai H, Yamamoto M, Murai Y, Watanabe T, et al. Combined light chain crystalline tubulopathy, podocytopathy, and histiocytosis associated with Bence-Jones κ protein diagnosed via immuno-electron microscopy. CEN case reports. 2021 Aug;10(3):453–8.

14. Kilic I, Picken MM, Velankar MM, Pambuccian SE. Bone marrow imprints of crystal-storing histiocytosis. Diagn Cytopathol. 2020 Mar;48(3):244–52.

15. Zizzo M, De Marco L, Zanelli M, Annessi V, Manenti A, Ascani S, et al. Localized Crystal-Storing Histiocytosis Involving Lower Rectum. Int J Surg Pathol. 2020 Jun;28(4):415–6.

16. Joo M, Kim NH. Gastric crystal-storing histiocytosis with concomitant mucosa-associated lymphoid tissue lymphoma. J Pathol Transl Med. 2020 Jul;54(4):332–5.

17. Wu P-S. Pulmonary crystal-storing histiocytosis: a case report and literature review. Int J Clin Exp Pathol. 2020;13(8):2169–72.

18. Contejean A, Larousserie F, Bouscary D, Dohan A, Deau-Fischer B, Szwebel TA, et al. A colonic mass revealing a disseminated crystal storing histiocytosis secondary to indolent multiple myeloma: A case report with literature review. Vol. 20, BMC Gastroenterology. BMC Gastroenterol; 2020.

19. Beltran M, Khurana S, Gil Y, Lewis JT, Kumar R, Foran JM. Nonimmunoglobulin Crystal-Storing Histiocytosis (CSH): Case Report and Literature Review. Case Rep Hematol. 2020 Oct;2020:1–4.

20. Reeders J, Arnold C, Chen J, Kirwan P, Lynnhtun K. Crystal-storing histiocytosis leading to the identification of IgG-kappa secreting lymphoplasmacytic lymphoma with crystalline nephropathy. Vol. 52, Pathology. Pathology; 2020. p. 283–6.

21. Braunstein MJ, Petrova-Drus K, Rosenbaum CA, Jayabalan DS, Rossi AC, Salvatore S, et al. Plasma Cell Myeloma Presenting with Amyloid-Laden Crystal-Negative Histiocytosis. Am J Clin Pathol. 2020 Dec;154(6):767–75.

22. Riefolo M, Malvi D, Bertuzzi C, Sabattini E, Valente S, Pasquinelli G, et al. Intrahepatocellular crystal storing mimicking a clinical liver disease during monoclonal gammopathy: report of a case and review of the literature. Ultrastruct Pathol. 2020 Jan;44(1):153–7.

23. Hafeez S, Shen P, Cho WC. Crystal-storing histiocytosis with plasma cell neoplasm in the setting of chronic carbamazepine exposure. J Pathol Transl Med. 2019 Mar;53(2):142–4.

24. Galeano-Valle F, Díaz-Crespo FJ, Melero-Martín R, Apaza-Chávez JE, Del-Toro-Cervera J, Demelo-Rodríguez P. Massive generalized crystal-storing histiocytosis associated with extracellular crystalline nephropathy: clinical, immunohistochemical, and ultrastructural studies of a unique disorder and review of the literature. CEN case reports. 2019 Aug;8(3):166–72.

25. Michon A, Cohen Aubart F, Haroche J, Charlotte F, Maksud P, Amoura Z. Long-bones involvement in generalized crystal-storing histiocytosis. Vol. 86, Joint Bone Spine. Joint Bone Spine; 2019. p. 652–3.

26. Tomsula J, Meis JM, Koy RD, Monheit J, Zieske A, Ro J, et al. Crystal storing histiocytosis: Unusual clinical presentations in two patients. Vol. 40, Annals of Diagnostic Pathology. Ann Diagn Pathol; 2019. p. 13–7.

27. Gupta RK, Rosenberg AZ, Bagnasco SM, Arend LJ. Renal crystal-storing histiocytosis involving glomeruli – A comprehensive clinicopathologic analysis. Ann Diagn Pathol. 2019 Dec;43.

28. Jaitly V, Hu Z, Ayala G, Wahed MA, Nguyen ND, Brown RE. M2 Macrophages in Crystal Storing Histiocytosis Associated with Plasma Cell Myeloma. Ann Clin Lab Sci. 2019 Sep;49(5):666–70.

29. Tao Q, Zhang W, Chen Z, Gao L, Yan J, Wang M, et al. Generalized crystal-storing histiocytosis with diffuse large B-cell lymphoma and monoclonal gammopathy in a Chinese elderly woman: A case report. BMC Cancer. 2019;

30. Rebecchini C, Trimeche M, Rosselet A, de Leval L. Crystal-Storing Histiocytosis. Vol. 27, International Journal of Surgical Pathology. Int J Surg Pathol; 2019. p. 399–400.

31. Fang H, Chiu A, Reichard KK. Crystal-storing histiocytosis in bone marrow: A clinicopathologic study of eight cases and review of the literature. Am J Clin Pathol. 2018;149(2):148–63.

32. Arnold CA, Frankel WL, Guo L, Krishnan C, Pfeil S, Schumacher M, et al. Crystal-storing Histiocytosis in the Stomach. Am J Surg Pathol. 2018 Oct;42(10):1317–24.

33. Flanagan ME, Keene CD, Louis DN, Juric-Sekhar G. Localized crystal-storing histiocytosis of the posterior fossa. Neuropathology. 2018 Oct;38(5):529–34.

34. Boudhabhay I, Titah C, Talbot A, Harel S, Verine J, Touchard G, et al. Multiple myeloma with crystal-storing histiocytosis, crystalline podocytopathy, and light chain proximal tubulopathy, revealed by retinal abnormalities: A case report. Med (United States). 2018 Dec;97(52).

35. Balakrishna JP, Jaffe ES. Crystal-storing histiocytosis associated with thymic extranodal marginal zone lymphoma. Blood. 2017 Oct;130(14):1683.

36. Uthamalingam P, Mehta S. Crystal-Storing Histiocytosis: Report of a Rare Case Presenting with Pathological Fracture of Femur. Is There More to the Entity? Int J Surg Pathol. 2017 Aug;25(5):458–61.

37. Wu CK, Yang AH, Lai HC, Lin BS. Combined proximal tubulopathy, crystal-storing histiocytosis, and cast nephropathy in a patient with light chain multiple myeloma. BMC Nephrol. 2017 May;18(1).

38. Kokuho N, Terasaki Y, Kunugi S, Onda N, Urushiyama H, Terasaki M, et al. Localized pulmonary crystal-storing histiocytosis complicating pulmonary mucosa-associated lymphoid tissue lymphoma presenting with multiple mass lesions. Hum Pathol. 2017 Jul;65:180–6.

39. Shah S, Sethi S, Arend L, Geetha D. Crystal-storing histiocytosis. Kidney Int. 2016 Feb;89(2):507.

40. Balakrishna J, Chen A, Urken M. Crystal storing histiocytosis clinically mimicking metastatic carcinoma: Report of a case and reviews of literature. Vol. 38, Head and Neck. Head Neck; 2016. p. E95–8.

41. Kanagal-Shamanna R, Xu-Monette ZY, Miranda RN, Dogan A, Zou D, Luthra R, et al. Crystal-storing histiocytosis: A clinicopathological study of 13 cases. Histopathology. 2016;

42. Szeto W, Garcia-Buitrago MT, Abbo L, Rosenblatt JD, Moshiree B, Morris MI. Clofazimine enteropathy: A rare and underrecognized complication of mycobacterial therapy. Open Forum Infect Dis. 2016 May;3(3).

43. Kaur A, Sethi S. Histiocytic and Nonhistiocytic Glomerular Lesions: Foam Cells and Their Mimickers. Am J Kidney Dis. 2016 Feb;67(2):329–36.

44. Li JJ, Henderson C. Cutaneous crystal storing histiocytosis: A report of two cases. J Cutan Pathol. 2015 Feb;42(2):136–43.

45. S L, JD K. Unusual breast mass: Lymphoma with crystal-storing histiocytosis. Blood. 2015 Apr;125(15):2445.

46. Lee JS, Im K, Park SN, Park HS, Kim JA, Choi Q, et al. A challenging diagnosis: Crystal-storing histiocytosis in plasma cell myeloma. Am J Clin Pathol. 2015 Feb;143(2):300–4.

47. Mittal R, Damato B, Coupland SE. Conjunctival extranodal marginal zone B-cell lymphoma with crystal-storing histiocytosis. Vol. 93, Acta Ophthalmologica. Acta Ophthalmol; 2015. p. e602–3.

48. Woehrer A, Kovacs GG. Clinical Neuropathology image 1-2015: Crystal-storing histiocytosis of the central nervous system. Clin Neuropathol. 2015;34(1):4–5.

49. Aline-Fardin A, Bender S, Fabiani B, Buob D, Brahimi S, Verpont MC, et al. Pseudo-peritoneal carcinomatosis presentation of a crystal-storing histiocytosis with an unmutated monoclonal k light chain. Med (United States). 2015 Aug;94(32).

50. Baird SM, Kenealy MK, Hoy R. Complete remission of Waldenström's associated generalized crystal-storing histiocytosis of IgM lambda subtype with bortezomib-based combination chemotherapy. Leuk Lymphoma. 2015 Nov;56(11):3233–5.

51. Tahara K, Miyajima K, Ono M, Sugio Y, Yamamoto I, Tamiya S. Crystal-storing histiocytosis associated with marginal-zone lymphoma. Jpn J Radiol. 2014;32(5):296–301.

52. Saluja K, Thakral B, Eldibany M, Goldschmidt R. Crystal storing histiocytosis associated with marginal zone B-cell lymphoma: A rare initial clinical presentation diagnosed by fine-needle

aspiration. Cytojournal. 2014 Jan;11(1).

53. Thakral B, Courville E. Crystal-storing histiocytosis with IgD k-associated plasma cell neoplasm. Vol. 123, Blood. Blood; 2014. p. 3540.

54. Radhakrishnan S, Maneksha V, Adulkar N. Crystal-storing histiocytosis masquerading ocular adnexal lymphoma: A case report and review of literature. Ophthal Plast Reconstr Surg. 2014;30(3).

55. Chaudhary S, Navarro M, Laser J, Berman E, Bhuiya T. Localized crystal-storing histiocytosis presenting as a breast nodule: An unusual presentation of a rare entity. Breast J. 2014;20(5):539–42.

56. Tsuji T, Yamasaki H, Hirano T, Toyozumi Y, Arima N, Tsuda H. Crystal-storing histiocytosis complicating marginal zone B-cell lymphoma of mucosa-associated lymphoid tissue. Vol. 100, International Journal of Hematology. Int J Hematol; 2014. p. 519–20.

57. Orr BA, Gallia GL, Dogan A, Rodriguez FJ. IgA/Kappa-restricted crystal storing histiocytosis involving the central nervous system characterized by proteomic analysis. Clin Neuropathol. 2014 Jan;33(1):23–8.

58. Vaid A, Caradine KD, Lai KK, Rego R. Isolated gastric crystal-storing histiocytosis: A rare marker of occult lymphoproliferative disorders. Vol. 67, Journal of Clinical Pathology. J Clin Pathol; 2014. p. 740–1.

59. Zhang C, Myers JL. Crystal-storing histiocytosis complicating primary pulmonary marginal zone lymphoma of mucosa-associated lymphoid tissue. Vol. 137, Archives of pathology & laboratory medicine. 2013. p. 1199–204.

60. Yano Y, Nagahama T, Matsui T, Chuman K, Takeichi M, Hirai F, et al. Gastric crystal-storing histiocytosis detected with asymptomatic Sjögren's syndrome: Report of a case and summary. Clin J Gastroenterol. 2013;6(3):237–42.

61. Johnson M, Mazariegos J, Lewis Pj, Pomakova D. Crystal storing histiocytosis presenting as a temporal lobe mass lesion. Surg Neurol Int. 2013;4(1):112.

62. Yu SC, Yao M, Liao SL. Crystal-storing histiocytosis in a patient with ocular extranodal marginal zone lymphoma. Vol. 160, British Journal of Haematology. Br J Haematol; 2013. p. 419–419.

63. Kawano N, Beppu K, Oyama M, Himeji D, Yoshida S, Kuriyama T, et al. Successful surgical treatment for pulmonary crystal-storing histiocytosis following the onset of gastric non-hodgkin lymphoma. J Clin Exp Hematop. 2013;53(3):241–5.

64. Miura TES, Takihi IY, Maekawa YH, Chauffaille M de LLF, Rizzatti EG, Sandes AF. Iron staining in gammopathy-related crystal-storing histiocytosis: A misleading feature to the differential diagnosis with Gaucher's disease. Vol. 110, Molecular Genetics and Metabolism. Mol Genet Metab; 2013. p. 414–5.

65. Duquesne A, Werbrouck A, Fabiani B, Denoyer A, Cervera P, Verpont MC, et al. Complete remission of monoclonal gammopathy with ocular and periorbital crystal storing histiocytosis and Fanconi syndrome. Hum Pathol. 2013 May;44(5):927–33.

66. Zardawi IM, Szabo F. Monoclonal plasma cell proliferation associated with crystal-storing histiocytosis on a background of plasmacytoid dendritic cell tumour in a patient with stable chronic myelomonocytic leukaemia. Vol. 62, Histopathology. Histopathology; 2013. p. 967–72.

67. Rossi G, De Rosa N, Cavazza A, Mengoli MC, Della Casa G, Nannini N, et al. Localized pleuropulmonary crystal-storing histiocytosis: 5 cases of a rare histiocytic disorder with

variable clinicoradiologic features. Am J Surg Pathol. 2013 Jun;37(6):906–12.

68. Singh H, Azad K, Kaur K. Clofazimine-induced enteropathy in a patient of leprosy. Indian J Pharmacol. 2013 Mar;45(2):197–8.

69. Dogan S, Barnes L, Cruz-Vetrano WP. Crystal-Storing Histiocytosis: Report of a Case, Review of the Literature (80 Cases) and a Proposed Classification. Head Neck Pathol. 2012 Mar;6(1):111–20.

70. Costanzi C, Bourdette D, Parisi JE, Woltjer R, Rodriguez F, Steensma D, et al. Crystal-storing histiocytosis: An unusual relapsing inflammatory CNS disorder. Mult Scler Relat Disord. 2012 Apr;1(2):95–9.

71. Da Cruz Perez DE, Silva-Sousa YTC, De Andrade BAB, Rizo VHT, Almeida LY, León JE, et al. Crystal-storing histiocytosis: A rare lesion in periapical pathology. Ann Diagn Pathol. 2012 Dec;16(6):527–31.

72. Ko HM, Da Cunha Santos G, Boerner SL, Bailey DJ, Geddie WR. Negative images of crystalline immunoglobulin in crystal storing histiocytosis: A potential cytologic mimic of mycobacteria in smears. Diagn Cytopathol. 2012 Oct;40(10):916–9.

73. Hu X, Liu J, Bai C, Wang J, Song X. Bortezomib combined with thalidomide and dexamethasone is effective for patient with crystal-storing histiocytosis associated with monoclonal gammopathy of undermined significance. Vol. 89, European Journal of Haematology. Eur J Haematol; 2012. p. 183–4.

74. Kaminsky IA, Wang AM, Olsen J, Schechter S, Wilson J, Olson R. Central nervous system crystal-storing histiocytosis: Neuroimaging, neuropathology, and literature review. Am J Neuroradiol. 2011 Feb;32(2).

75. Khurram SA, McPhaden A, Hislop WS, Hunter KD. Crystal storing histiocytosis of the tongue as the initial presentation of multiple myeloma. Oral Surgery, Oral Med Oral Pathol Oral Radiol Endodontology. 2011 Apr;111(4):494–6.

76. Lesesve JF, Bronowicki JP, Galed-Placed I. Crystal-storing histiocytosis in ascites from a patient with IgM kappa lymphoplasmacytic lymphoma. Vol. 22, Cytopathology. Cytopathology; 2011. p. 207–8.

77. Gao FF, Khalbuss WE, Austin RM, Monaco SE. Cytomorphology of crystal storing histiocytosis in the breast associated with lymphoma: A case report. Acta Cytol. 2011;55(3):302–6.

78. Qureshi A, Kashif M. Crystal-storing histiocytosis. Blood. 2010 Apr;115(13):2568.

79. Yang Y, Bekeris LG, Vogl DT, Bagg A. Crystal-storing histiocytosis in plasma cell myeloma. Am J Hematol. 2010 Jun;85(6):444–5.

80. Rossi G, Morandi U, Nannini N, Fontana G, Pifferi M, Casali C. Crystal-storing histiocytosis presenting with pleural disease Correspondence. Vol. 56, Histopathology. Histopathology; 2010. p. 403–5.

81. El Hamel C, Thierry A, Trouillas P, Bridoux F, Carrion C, Quellard N, et al. Crystal-storing histiocytosis with renal Fanconi syndrome: Pathological and molecular characteristics compared with classical myeloma-associated Fanconi syndrome. Nephrol Dial Transplant. 2010 Sep;25(9):2982–90.

82. Todd WU, Drabick JJ, Benninghoff MG, Frauenhoffer EE, Zander DS. Pulmonary crystal-storing histiocytosis diagnosed by computed tomography-guided fine-needle aspiration. Diagn Cytopathol. 2010 Apr;38(4):274–8.

83. Alayed KM, Alabdulaali MK, Alkhairy KS, Elnour S, Alhajjaj A. Aggressive systemic mastocytosis with CharcotLeyden crystals-associated crystal storing histiocytosis in bone marrow. Pathology. 2010;42(1):85–7.

84. Kurabayashi A, Iguchi M, Matsumoto M, Hiroi M, Kume M, Furihata M. Thymic mucosa-associated lymphoid tissue lymphoma with immunoglobulin- storing histiocytosis in Sjögren's syndrome: Case Report. Pathol Int. 2010 Feb;60(2):125–30.

85. Sailey CJ, Alexiev BA, Gammie JS, Pinell-Salles P, Stafford JL, Burke A. Crystal-storing histiocytosis as a cause of symptomatic cardiac mass: Histologic, immunohistochemical, and electron microscopic findings. Arch Pathol Lab Med. 2009 Nov;133(11):1861–4.

86. De Alba Campomanes AG, Rutar T, Crawford JB, Seiff S, Goodman D, Grenert J. Crystal-storing histiocytosis and crystalline keratopathy caused by monoclonal gammopathy of undetermined significance. Cornea. 2009 Oct;28(9):1081–4.

87. Farooq U, Bayerl MG, Abendroth CS, Verma N, Talamo G. Renal crystal storing histiocytosis in a patient with multiple myeloma. Vol. 88, Annals of Hematology. Ann Hematol; 2009. p. 807–9.

88. Lee WS, Kim SR, Moon H, Choe YH, Park SJ, Lee HB, et al. Pulmonary crystal-storing histiocytoma in a patient without a lymphoproliferative disorder. Am J Med Sci. 2009;338(5):421–4.

89. László R, Degrell P, Kellermayer M, Bollmann D, Egyed M, Seres L, et al. Crystal-storing histiocytosis associated with only one of two consecutive, but genetically unrelated B-cell lymphomas. Pathol Res Pract. 2009 Apr;205(4):273–8.

90. Kar R, Dutta S, Bhargava R, Tyagi S. Crystal storing histiocytosis: A rare presentation of plasma cell myeloma. Indian J Hematol Blood Transfus. 2008;24(2):63–6.

91. Keane C, Gill D. Multi-organ involvement with crystal-storing histiocytosis. Br J Haematol. 2008 Jun;141(6):750.

92. Rodriguez FJ, Gamez JD, Vrana JA, Theis JD, Giannini C, Scheithauer BW, et al. Immunoglobulin derived depositions in the nervous system: Novel mass spectrometry application for protein characterization in formalin-fixed tissues. Lab Investig. 2008 Oct;88(10):1024–37.

93. Bodi I, Hortobágyi T, Buk S. A 72-year-old woman with right frontal extra-axial mass. Brain Pathol. 2008 Apr;18(2):279–82.

94. Kusakabe T, Watanabe K, Mori T, Iida T, Suzuki T. Crystal-storing histiocytosis associated with MALT lymphoma of the ocular adnexa: A case report with review of literature. Virchows Arch. 2007 Jan;450(1):103–8.

95. Joo M, Kwak JE, Chang SH, Kim H, Chi JG, Moon YS, et al. Localized gastric crystal-storing histiocytosis [2]. Vol. 51, Histopathology. Histopathology; 2007. p. 116–8.

96. Lewis JT, Candelora JN, Hogan RB, Briggs FR, Abraham SC. Crystal-storing histiocytosis due to massive accumulation of Charcot-Leyden crystals: A unique association producing colonic polyposis in a 78-year-old woman with eosinophilic colitis. Am J Surg Pathol. 2007 Mar;31(3):481–5.

97. Chantranuwat C. Noncrystallized form of immunoglobulin-storing histiocytosis as a cause of chronic lung infiltration in multiple myeloma. Ann Diagn Pathol. 2007 Jun;11(3):220–2.

98. Pock L, Stuchlík D, Hercogová J. Crystal storing histiocytosis of the skin associated with multiple myeloma. Int J Dermatol. 2006 Dec;45(12):1408–11.

99. De Lastours V, Papo T, Cazals-Hatem D, Eden A, Feydy A, Belmatoug N, et al. Bone involvement in generalized crystal-storing histiocytosis. J Rheumatol. 2006 Nov;33(11):2354–8.

100. Stokes MB, Aronoff B, Siegel D, D'Agati VD. Dysproteinemia-related nephropathy associated with crystal-storing histiocytosis. Kidney Int. 2006 Aug;70(3):597–602.

101. Fairweather PM, Williamson R, Tsikleas G. Pulmonary extranodal marginal zone lymphoma with massive crystal storing histiocytosis. Am J Surg Pathol. 2006 Feb;30(2):262–7.

102. Galed-Placed I. Immunoglobulin crystal-storing histiocytosis in a pleural effusion from a woman with Iga κ multiple myeloma: A case report. Acta Cytol. 2006;50(5):539–41.

103. Pitman SD, Wang J, Serros ER, Zuppan C. A 70-year-old woman with acute renal failure. Arch Pathol Lab Med. 2006 Jul;130(7):1077–8.

104. Weichman K, Dember LM, Prokaeva T, Wright DG, Quillen K, Rosenzweig M, et al. Clinical and molecular characteristics of patients with non-amyloid light chain deposition disorders, and outcome following treatment with high-dose melphalan and autologous stem cell transplantation. Bone Marrow Transplant. 2006 Sep;38(5):339–43.

105. Ionescu DN, Pierson DM, Qing G, Li M, Colby T V., Leslie KO. Pulmonary crystal-storing histiocytoma. Arch Pathol Lab Med. 2005 Sep;129(9):1159–63.

106. Tholouli E, Krebs M, Reeve R, Houghton JB. Crystal-storing histiocytosis in a patient with IgGκ multiple myeloma. Br J Haematol. 2005 Feb;128(4):412.

107. Pais A V., Pereira S, Garg I, Stephen J, Antony M, Inchara YK. Intra-abdominal, crystal-storing histiocytosis due to clofazimine in a patient with lepromatous leprosy and concurrent carcinoma of the colon. Lepr Rev. 2004 Jun;75(2):171–6.

108. Papla B, Spólnik P, Rzenno E, Zduńczyk A, Rudzki Z, Okoń K, et al. Generalized crystal-storing histiocytosis as a presentation of multiple myeloma: A case with a possible pro-aggregation defect in the immunoglobulin heavy chain. Virchows Arch. 2004 Jul;445(1):83–9.

109. Zioni F, Giovanardi P, Bozzoli M, Artusi T, Bonacorsi G, Sighinolfi P. Massive bone marrow crystal-storing histiocytosis in a patient with IgA-lambda multiple myeloma and extensive extramedullary disease. A case report. Tumori. 2004;90(3):348–51.

110. Sun Y, Tawfiqul B, Valderrama E, Kline G, Kahn LB. Pulmonary crystal-storing histiocytosis and extranodal marginal zone B-cell lymphoma associated with a fibroleiomyomatous hamartoma. Ann Diagn Pathol. 2003;7(1):47–53.

111. Sethi S, Cuiffo BP, Pinkus GS, Rennke HG. Crystal-storing histiocytosis involving the kidney in a low-grade B-cell lymphoproliferative disorder. Am J Kidney Dis. 2002;39(1):183–8.

112. Lebeau A, Zeindl-Eberhart E, Müller EC, Müller-Höcker J, Jungblut PR, Emmerich B, et al. Generalized crystal-storing histiocytosis associated with monoclonal gammopathy: Molecular analysis of a disorder with rapid clinical course and review of the literature. Blood. 2002 Sep;100(5):1817–27.

113. Coupland SE, Foss HD, Hummel M, Stein H. Extranodal marginal zone B-cell lymphoma of the lacrimal gland associated with crystal-storing histiocytosis. Ophthalmology. 2002;109(1):105–10.

114. Wada R, Ebina Y, Kurotaki H, Yagihashi S. Intracytoplasmic immunoglobulin crystals in follicular lymphoma. Hum Pathol. 2002 Nov;33(11):1141–4.

115. Robak T, Urbańska-Ryś H, Jerzmanowski P, Bartkowiak J, Liberski P, Kordek R. Lymphoplasmacytic lymphoma with monoclonal gammopathy-related Pseudo-Gaucher cell infiltration in bone marrow and spleen - Diagnostic and therapeutic dilemmas. Leuk Lymphoma. 2002 Dec;43(12):2343–50.

116. Sukpanichnant S, Hargrove NS, Kachintorn U, Manatsathit S, Chanchairujira T, Siritanaratkul N, et al. Clofazimine-induced crystal-storing histiocytosis producing chronic abdominal pain in a leprosy patient. Am J Surg Pathol. 2000 Jan;24(1):129–35.

117. Thorson P, Hess JL. Transformation of monocytoid B-cell lymphoma to large cell lymphoma associated with crystal-storing histiocytes. Arch Pathol Lab Med. 2000;124(3):460–2.

118. Jones D, Bhatia VK, Krausz T, Pinkus GS. Crystal-storing histiocytosis: A disorder occurring in plasmacytic tumors expressing immunoglobulin kappa light chain. Hum Pathol. 1999;30(12):1441–8.

119. Prasad ML, Charney DA, Sarlin J, Keller SM. Pulmonary immunocytoma with massive crystal storing histiocytosis: A case report with review of literature. Am J Surg Pathol. 1998 Sep;22(9):1148–53.

120. García JF, Sánchez E, Lloret E, Martín J, Piris MA. Crystal-storing histiocytosis and immunocytoma associated with multifocal fibrosclerosis. Histopathology. 1998;33(5):459–64.

121. Bosman C, Camassei FD, Boldrini R, Piro FR, Saponara M, Romeo R, et al. Solitary crystal-storing histiocytosis of the tongue in a patient with rheumatoid arthritis and polyclonal hypergammaglobulinemia. Arch Pathol Lab Med. 1998 Oct;122(10):920–4.

122. Llobet M, Castro P, Barceló C, Trull JM, Campo E, Bernadó L. Massive crystal-storing histiocytosis associated with low-grade malignant B-cell lymphoma of MALT-type of the parotid gland. Diagn Cytopathol. 1997;17(2):148–52.

123. Harada M, Shimada M, Fukayama M, Kaneko T, Kitazume K, Weiss SW. Crystal-storing histiocytosis associated with lymphoplasmacytic lymphoma mimicking Weber-Christian disease: Immunohistochemical, ultrastructural, and gene-rearrangement studies. Hum Pathol. 1996;27(1):84–7.

124. Kaufmann O, Hansen A, Deicke P, Burmester GR, Dietel M. Subcutaneous crystal-storing histiocytosis associated with lymphoplasmacytic lymphoma (immunocytoma). Pathol Res Pract. 1996;192(11):1148–51.

125. Jones D, Renshaw AA. Recurrent crystal-storing histiocytosis of the lung in a patient without a clonal lymphoproliferative disorder. Arch Pathol Lab Med. 1996 Oct;120(10):978–80.

126. Friedman MT, Molho L, Valderrama E, Kahn LB. Crystal-storing histiocytosis associated with a lymphoplasmacytic neoplasm mimicking adult rhabdomyoma: A case report and review of the literature. Vol. 120, Archives of Pathology and Laboratory Medicine. Arch Pathol Lab Med; 1996. p. 1133–6.

127. Kapadia SB, Enzinger FM, Heffner DK, Hyams VJ, Frizzera G. Crystal-storing histiocytosis associated with lymphoplasmacytic neoplasms: Report of three cases mimicking adult rhabdomyoma. Am J Surg Pathol. 1993;17(5):461–7.

128. Yamamoto T, Hishida A, Honda N, Ito I, Shirasawa H, Nagase M. Crystal-storing histiocytosis and crystalline tissue deposition in multiple myeloma. Arch Pathol Lab Med. 1991;115(4):351–4.

129. Takahashi K, Naito M, Takatsuki K, Kono F, Chitose M, Ooshima S, et al. MULTIPLE MYELOMA,

IgA x TYPE, ACCOMPANYING CRYSTAL-STORING HISTIOCYTOSIS AND AMYLOIDOSIS. Pathol Int. 1987;37(1):141–54.

130. Hashimoto N, Kurihara K, Sakai H. Extramedullary plasmacytoma with crystal inclusions arising from the palatal tonsil. J Oral Pathol Med. 1983;12(5):309–18.

131. Padmalatha C, Warner TFCS, Hafez GR. Pseudo-gaucher cell in IgMk plasmacytoid lymphoma. Am J Surg Pathol. 1981;5(5):501–5.

132. Mullen B, Chalvardjian A. Crystalline tissue deposits in a case of multiple myeloma. Arch Pathol Lab Med. 1981;105(2):94–7.

133. Rao NA, Font RL. Plasmacytic Conjunctivitis With Crystalline Inclusions: Immunohistochemical and Ultrastructural Studies. Arch Ophthalmol. 1980;98(5):836–41.

134. Addis BJ, Isaacson P, Billings JA. Plasmacytoma of lymph nodes. Cancer. 1980;46(2):340–6.

135. Scullin DC, Shelburne JD, Cohen HJ. Pseudo-Gaucher cells in multiple myeloma. Am J Med. 1979;67(2):347–52.

136. Terashima K, Takahashi K, Kojima M, Imai Y, Tsuchida S, Migita S, et al. KAPPA-TYPE LIGHT CHAIN CRYSTAL STORAGE HISTIOCYTOSIS. Pathol Int. 1978;28(1):111–38.

137. Pinkerton RM, Robertson DM. Corneal and conjunctival changes in dysproteinemia. Invest Ophthalmol. 1969;8(4):357–64.

138. Glaus A. Über multiples Myelozytom mit eigenartigen, zum Teil kristallähnlichen Zelleinlagerungen, kombiniert mit Elastolyse und ausgedehnter Amyloidose und Verkalkung. Virchows Arch Pathol Anat Physiol Klin Med. 1917 Apr;223(3):301–39.
